# Zika virus-induces metabolic alterations in fetal neuronal progenitors that could influence in neurodevelopment during early pregnancy

**DOI:** 10.1242/bio.059889

**Published:** 2023-04-24

**Authors:** Javier Gilbert-Jaramillo, Ujang Purnama, Zoltán Molnár, William S. James

**Affiliations:** ^1^ James & Lillian Martin Centre, Sir William Dunn School of Pathology, University of Oxford, South Parks Road, Oxford OX1 3RE, UK; ^2^ Department of Physiology, Anatomy and Genetics, Sherrington Building, University of Oxford, Parks Road, Oxford OX1 3PT, UK; ^3^ ESPOL Polytechnic University, Escuela Superior Politécnica del Litoral, ESPOL, Facultad de Ciencias de la Vida, Campus Gustavo Galindo Km. 30.5 Vía Perimetral, P.O. Box 09-01-5863, Guayaquil, Ecuador

**Keywords:** ZIKV, Zika virus, Fetal neurodevelopment, Metabolism, Neurometabolism, Neuronal progenitors

## Abstract

Cortical development consists of an orchestrated process in which progenitor cells exhibit distinct fate restrictions regulated by time-dependent activation of energetic pathways. Thus, the hijacking of cellular metabolism by Zika virus (ZIKV) to support its replication may contribute to damage in the developing fetal brain. Here, we showed that ZIKV replicates differently in two glycolytically distinct pools of cortical progenitors derived from human induced pluripotent stem cells (hiPSCs), which resemble the metabolic patterns of quiescence (early hi-NPCs) and immature brain cells (late hi-NPCs) in the forebrain. This differential replication alters the transcription of metabolic genes in both pools of cortical progenitors but solely upregulates the glycolytic capacity of early hi-NPCs. Analysis using Imagestream^®^ revealed that, during early stages of ZIKV replication, in early hi-NPCs there is an increase in lipid droplet abundance and size. This stage of ZIKV replication significantly reduced the mitochondrial distribution in both early and late hi-NPCs. During later stages of ZIKV replication, late hi-NPCs show reduced mitochondrial size and abundance. The finding that there are alterations of cellular metabolism during ZIKV infection which are specific to pools of cortical progenitors at different stages of maturation may help to explain the differences in brain damage over each trimester.

## Teaser

**Summary:** Distinct metabolic alterations in neuronal progenitors at different stages of maturation during ZIKV infection may explain the pathophysiology over each trimester and highlight a therapeutic potential of metabolic remodelling.

## INTRODUCTION

Intrauterine development during the first trimester of pregnancy includes a series of orchestrated cellular processes, mainly cell division and differentiation, which give origin to primitive tissue and organs (Burton et al., 2001). The brain develops from a neural plate that forms a tube. The inner lining of this tube contains the germinal zone where most of the progenitor cells are situated (Molnár et al., 2019). Neuronal progenitor cells at different stages of proliferation and differentiation exhibit specific activation and shifts between the main cellular metabolic pathways (glycolysis, glucose and, fatty acid oxidation; Zheng et al., 2016; Knobloch et al., 2022, 2017). The shift from cytosolic glucose metabolism to mitochondrial glucose oxidation and the activation of fatty acid oxidation are thought to signal for differentiation of quiescent neuronal progenitors to proliferative neuronal progenitors (Knobloch and Jessberger, 2017).

Maternal malnutrition during pregnancy may alter the nutrient supply and metabolism of the fetal brain imposing severe consequences to normal development (Burton et al., 2001; Wu et al., 2004). These consequences are comparable and potentially contribute to those observed during infections with neurotropic viruses such as Zika virus (ZIKV) (Barbeito-Andrés et al., 2020). The maternal-fetal circulation allows ZIKV to reach the developing fetal brain where its infection and metabolic hijacking of brain cells potentially underpin the anatomical and physiological damage observed in newborns (Gilbert-Jaramillo et al., 2019; Rothan et al., 2019). This damage is mainly observed after infection during the first trimester (Gilbert-Jaramillo et al., 2019; Krow-Lucal et al., 2018) and is not observed to the same extent when maternal infection occurs during mid and late trimesters (Krow-Lucal et al., 2018; Sarno et al., 2016), after the accelerated fetal brain expansion has occurred.

There are few studies investigating whether ZIKV infection differs among brain progenitors at different stages of maturation (Ferraris et al., 2019) and no studies on how the distinct metabolic profile of these progenitors during differentiation may influence the specific brain damage over each trimester. Thus, we investigated the metabolic stress imposed by ZIKV infection in *in vitro* cultures of human induced pluripotent stem cell (hiPSCs) derived cortical neuronal progenitors (hi-NPCs) at different times under culture. These hi-NPC cultures contain similar ratios of different forebrain progenitors but differ on their metabolic profiles suggesting different maturation stages that may correlate with pools of cortical progenitors present over different trimesters. Here, we show that these pools of cortical progenitors differ in their rate of glucose and fatty acid metabolism and that this is differentially exploited by ZIKV. ZIKV infection shows a broad dysregulation of the transcriptional profile of genes involved in glucose metabolism, fatty acid oxidation and, fatty acid biosynthesis in hi-NPCs. Few changes in mitochondrial homeostasis are also observed in hi-NPCs yet, these changes are dependent on the maturation stage of hi-NPCs and linked to specific times during ZIKV replication. Notably, ZIKV increases the glycolytic capacity in quiescent-like progenitors (early hi-NPCs), which are characterised by a greater glucose metabolic rate compared to immature brain cells (late hi-NPCs). In similar fashion, few changes in the intracellular abundance and size of lipid droplets are observed exclusively in early hi-NPCs at early stages of ZIKV replication.

## RESULTS

### Differentiation of human cortical neuronal progenitors rescues metabolically distinct populations that recapitulate developmental stages of the forebrain

**Fig. 1.**
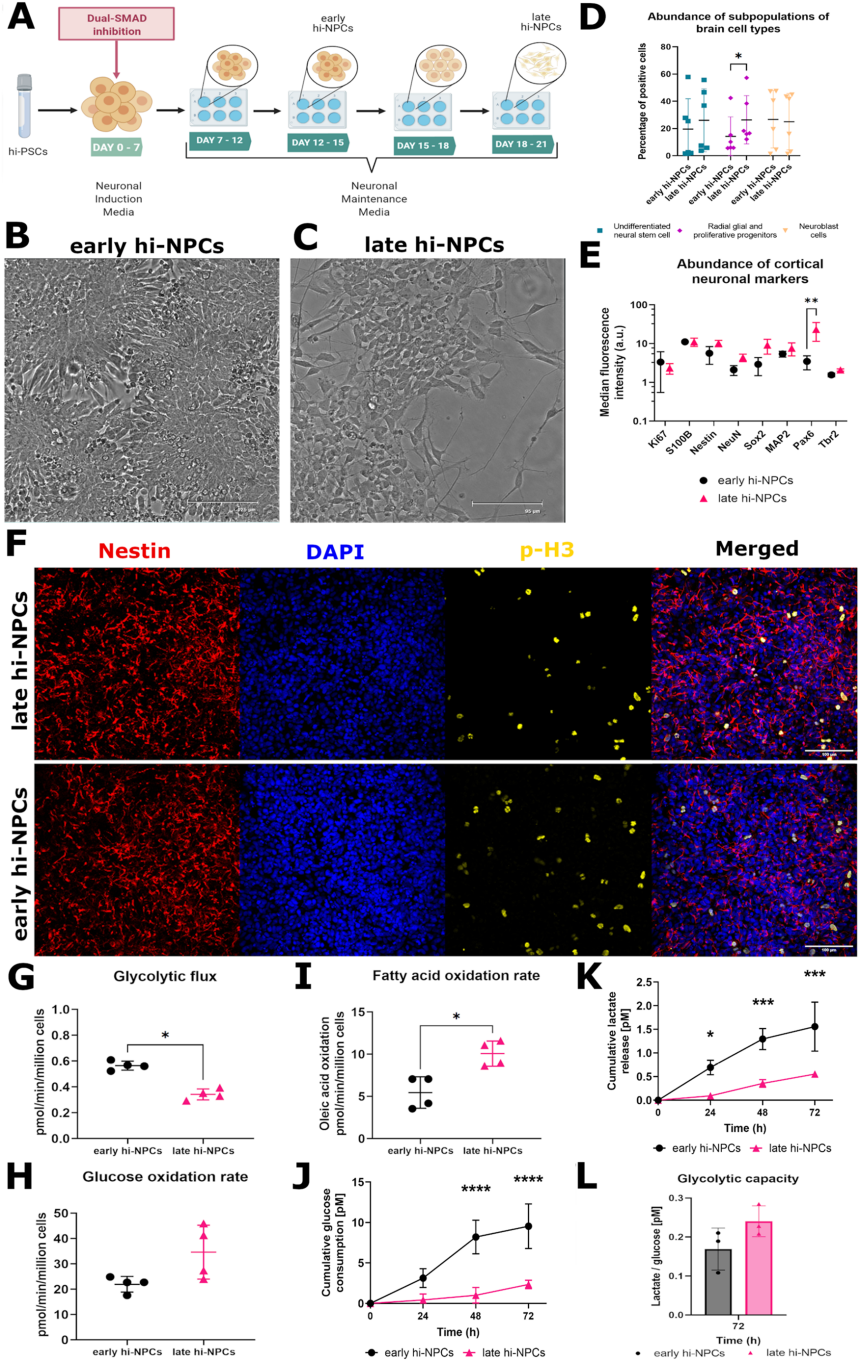
**hiPSC cortical differentiation produces forebrain progenitors at different stages of maturation.** (A) Schematic of 2D differentiation of hiPSCs to cortical neuronal progenitors (hi-NPCs). Brightfield images showing differences in morphology between early (B) and late hi-NPCs (C). Scale bars of 125 µm and 95 µm, respectively. (D) Dot plot showing the percentage summary of brain cell types in early and late hi-NPCs. *N*=1 cortical differentiation done in triplicates for each of three independent patients' lines. Significance was calculated by two-way ANOVA with Šidák's multiple comparisons post-hoc test. (C) Dot plot showing the relative median fluorescence intensity of a panel of conventional early cortical neuronal progenitor markers in early and late hi-NPCs. *n*=1 cortical differentiation of two independent patients' lines each conducted in four replicates. Significance was calculated by a mixed-effects model with Šidák correction. (F) Representative confocal images (10x) of the detection of markers of cellular proliferation phospho-Histone3 (p-H3) and of *in vitro* cortical neuronal progenitors nestin in early and late hi-NPCs. Scale bar: 100 µm. Intracellular radioactive tracing showing the metabolic remodelling of the (G) glycolytic flux, (H) glucose oxidation rate and, (I) fatty acid oxidation rate between early and late hi-NPCs. *n*=2 cortical differentiations each measured at two different time-points, 10 and 14 h post plating, in five replicates for a patient line. Significance was calculated by non-parametric two-tailed Mann–Whitney *U*-test. Dot plots showing the estimated cumulative glucose consumption (J) and lactate release (K) in hi-NPCs cultured over 72 h. (L) Bar graphs displaying the glycolytic capacity of per hi-NPC subtype at 72 h post-culture. *n*=1 cortical differentiation measured in triplicates for three independent patients' lines. Significance was calculated by two-way ANOVA with Šidák's multiple comparisons post-hoc test. Error bars display mean±s.d. Significance is shown when *P<0.05, **P<0.01, ***P<0.001, ****P<0.0001.

Differentiation of cortical neuronal progenitors (hi-NPCs) using a 2D adaptation from Shi et al. (2012), Robbins et al. (2018) (Fig. 1A), produces, at different time-points, hi-NPCs with distinct morphology (Fig. 1B and C). Based on the time under culture, these cells were called early and late hi-NPCs. Early hi-NPCs exhibited epithelia-like cells characterised by a large cytoplasm and size with few axonal/dendritic projections compared to late hi-NPCs. Early hi-NPC cultures are also characterised by cellular clusters forming rosette-like structures suggesting the presence of neuronal progenitor cells (Fig. S1), whilst late hi-NPCs exhibit cells with ‘star-shaped’ morphology. Cells within the culture of late hi-NPCs displayed abundant thin and ramified projections connecting distant cells within the dish (Fig. 1A and Fig. S1). Characterization conducted by flow cytometry detection of membrane-bound receptors showed that differentiation of early and late hi-NPCs cultures produced subpopulations of brain cell types (undifferentiated neural stem cells, radial glial and proliferative progenitors and, neuroblast cells) at similar ratios. These populations significantly differ (*P*≥0.0344) exclusively in the abundance of radial glial and proliferative progenitors between hi-NPCs cultures (Fig. 1D). Detection levels of expression of conventional morphological and proliferative markers by flow cytometry corroborated this data. There were no significant differences in the expression of most of the proteins of interest between the pools of cortical progenitors with Pax6 showing a significantly higher expression in late compared to early hi-NPCs (Fig. 1E). Positive staining of neuronal progenitors' markers was validated by confocal imaging (Fig. 1F and Fig. S2).

Cellular metabolism is crucial during differentiation of neuronal progenitor cells (Knobloch and Jessberger, 2017). We traced intracellular short-term metabolic fluxes (between 10- and 14-h post-passage) using radioactive-labelled substrates and found that the metabolic output from hi-NPC cultures differ from each other. Analysis of glucose processing showed a significantly lower glycolytic flux (*P*=0.0286) in late compared to early hi-NPCs (Fig. 1G) and no differences in the mitochondrial glucose oxidation (Fig. 1H). The estimation of mitochondrial lipid oxidation by measuring the oxidation rate of oleic acid showed to be significantly higher in late compared to early hi-NPCs (*P*=0.0286) (Fig. 1I). This was supported by the long-term tracing of glucose consumption and lactate release. Early hi-NPCs displayed a significantly greater consumption of glucose (*P*<0.0001) and lactate release (*P*<0.0037) over 72 h of culture (Fig. 1J and K). However, the glycolytic capacity, estimated as a ratio of pico Molar [pM] of lactate released per [pM] of glucose consumed per cell, showed not to be significantly different between hi-NPCs (Fig. 1L).

### ZIKV differentially replicates in cortical progenitors at different stages of maturation inducing specific alterations

**Fig. 2.**
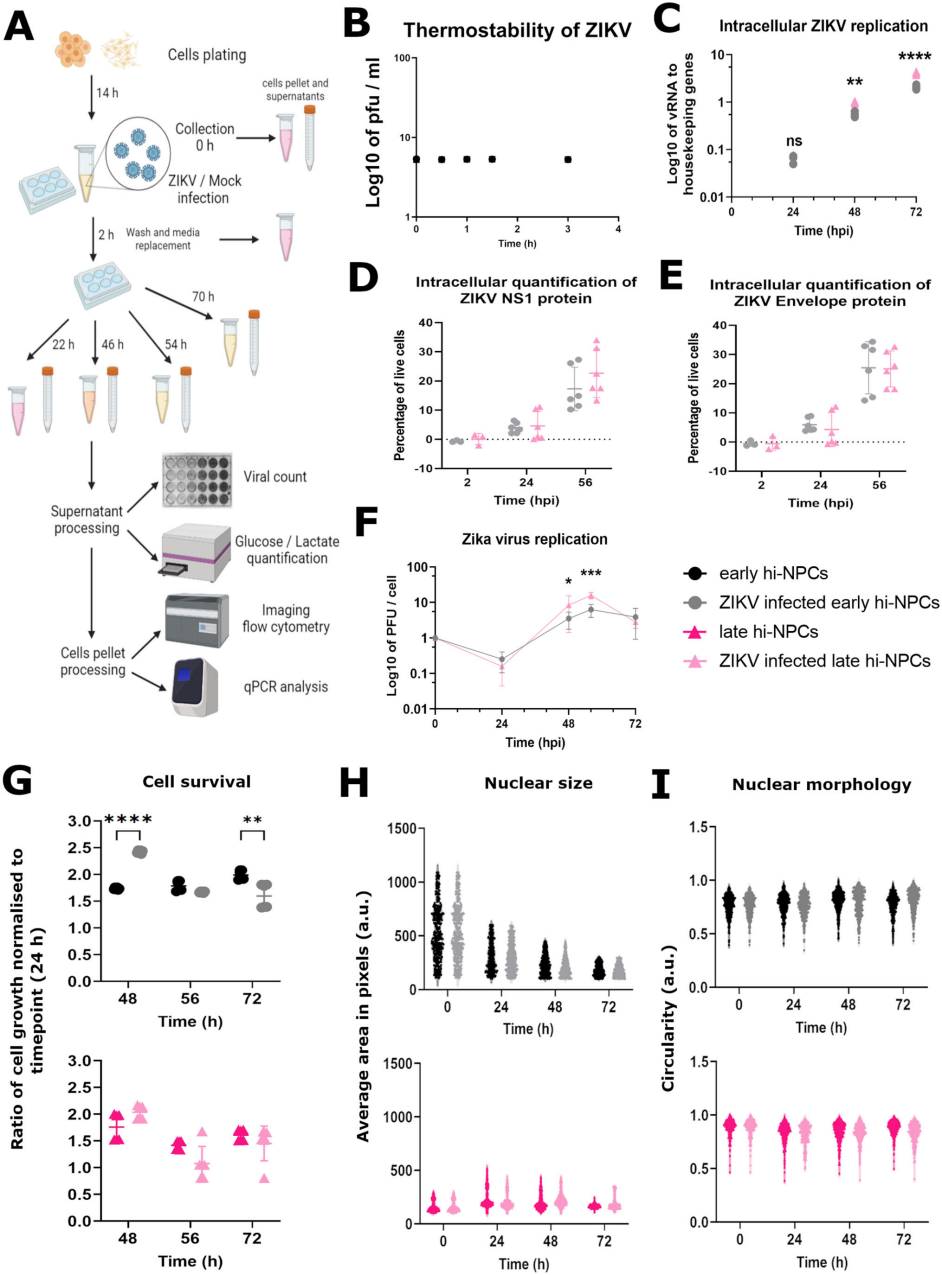
**ZIKV exhibits differential replication rates and cellular stress in cortical progenitors at different stages of maturation.** (A) Schematic 2D representation of the protocol conducted to investigate the effects of hi-NPCs exposure to ZIKV. (B) Dot plot showing the stability of ZIKV at 37°C. *n*=3 experiments titrated by plaque assay in triplicates. Significance was calculated by a mixed-effects model with Dunnett's multiple comparisons test. (C) Intracellular ZIKV replication relative to housekeeping genes. qPCR conducted in four replicates. Dots displaying the values of each patient line. *n*=1 viral infection conducted in duplicates of three independent patients' lines. Significance was calculated by two-way ANOVA with Šidák's multiple comparisons post-hoc test. Percentage of live cells containing (D) ZIKV NS1 protein a©(E) ZIKV Envelope protein. A minimum of 10,000 cells per patient line were processed by flow cytometry. (F) Dot plot showing extracellular release of ZIKV infectious particles by hi-NPCs. ZIKV quantification was done by plaque assay in triplicates. *n*=2 viral infection conducted in duplicates of three independent patients' lines. Significance was calculated by mixed-effects model with Šidák correction. (G) Dot plots showing the cell viability of hi-NPCs infected with ZIKV compared to non-infected controls. *n*=2 viral infection conducted in triplicates for each of the three patients' line. Significance was calculated by two-way ANOVA with Šidák's multiple comparisons. Violin plots displaying the mean value for ≥150 nuclei per condition. Nuclei stained with DAPI showing (H) nuclear size and (I) nuclear morphology of hi-NPCs. Significance was calculated by mixed-effects model with Šidák's multiple comparisons test. Error bars display mean±s.d. Significance is shown when *P<0.05, **P<0.01, ***P<0.001.

ZIKV infection in hi-NPCs at different stages of maturation may elucidate contributing mechanisms for the distinct brain damage induced by ZIKV infection over each trimester. To test this, we infected pools of cortical progenitors (early and late hi-NPCs; Fig. 2A) which metabolically recapitulate cells at different stages of maturation. Infections were conducted over 2 h at 37°C, time during which there was no significant thermal decay of the infectivity of ZIKV (Fig. 2B). Both early and late hi-NPCs showed susceptibility to ZIKV infection with differences in the replication rates but not in the initial viral uptake (Fig. 2F). RNA quantification showed that late hi-NPCs accumulate significantly more transcripts of ZIKV in a time-dependent manner than early hi-NPC (Fig. 2C). At 48 h.p.i., late hi-NPCs showed an increased ratio of 1.77 in intracellular copies of vRNA compared the early hi-NPCs. This further increased to a ratio of 1.98 at 72 h.p.i. (Fig. 2C). Analysis of the intracellular accumulation of the non-structural viral protein NS1 (Fig. 2D) and the virion-associated Envelope protein (Env; Fig. 2E) by flow cytometry revealed similar levels of intracellular ZIKV proteins between early and late hi-NPCs. Detection levels of NS1 compared to Env protein were also similar with ∼10% of the population infected at 24 h and ∼30% at 56 h. Significant accumulation of ZIKV transcripts in late hi-NPCs were translated into a significantly greater release of virions at 48 h.p.i. (late:early ratio of 2.38) and 56 h.p.i. (late:early ratio of 2.52) compared to early hi-NPCs (Fig. 2F).

Cell viability following ZIKV infection was calculated as the growth rate of hi-NPCs compared to their post-stimulation survival (cell number calculated at 24 h). Zika infection in early hi-NPCs significantly increased the rate of cellular proliferation at 48 h.p.i. (*P*≤0.0001) followed by a decrease rate at 56 and 72 h.p.i. At the latter timepoint, significant cell death was observed (*P*≤0.0031). In late hi-NPCs, no significant changes were calculated in the growth rate of infected compared to non-infected cells over 72 h (Fig. 2G).

In addition, ZIKV infection reflected changes in nuclear morphology that were distinct between cortical progenitors at different stages of maturation (Fig. S3). However, these changes were potentially exclusive to infected cells containing replicating ZIKV thus, measurements of nuclear damage represented as size and morphology within the mixed population of virus-containing and non-containing infected cells did not show significant differences between the non-infected and infected conditions (Fig. 2H and I).

### ZIKV infection increases the glycolytic capacity of undifferentiated cortical progenitors

**Fig. 3.**
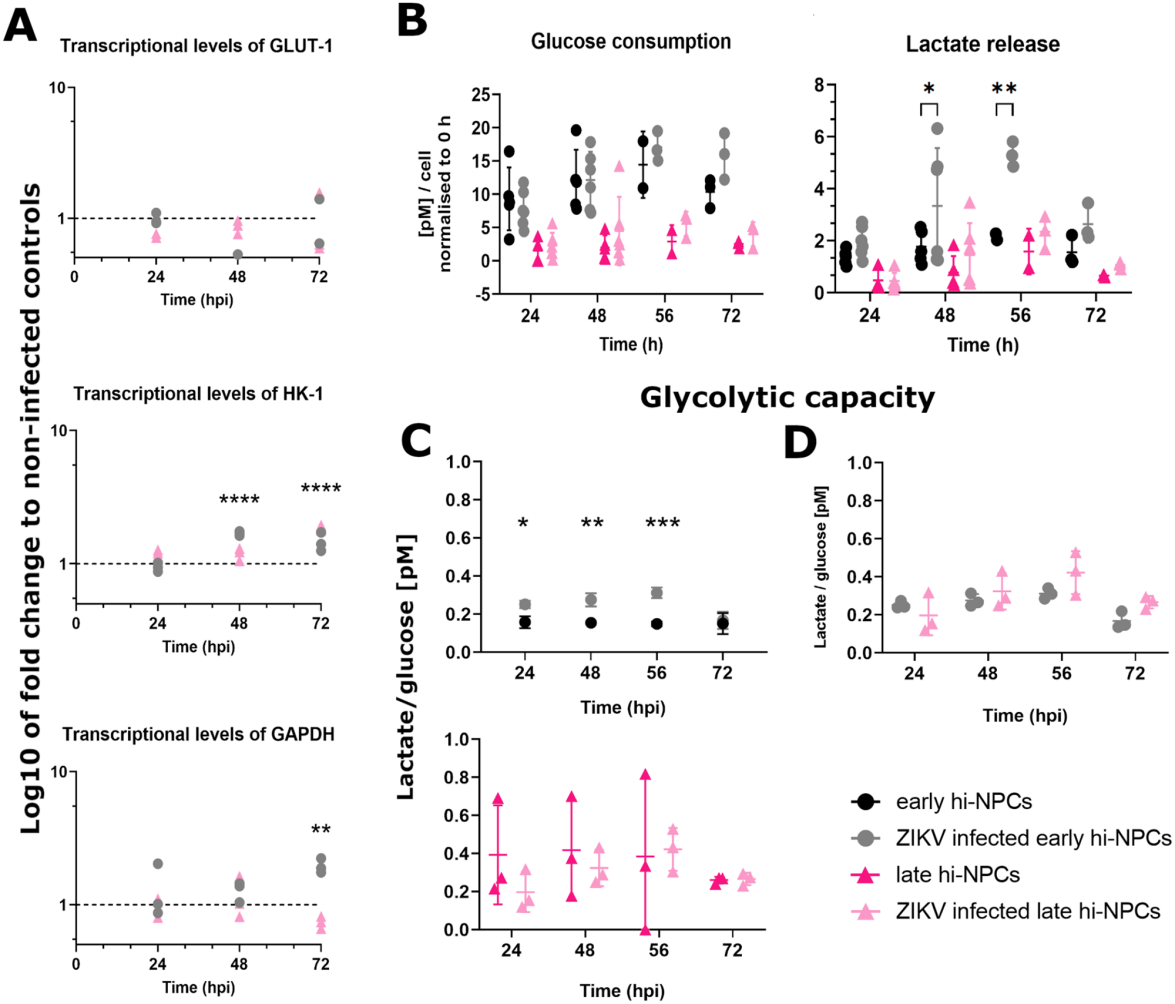
**ZIKV infection increments the glycolytic capacity of early but not late neuronal progenitors (hi-NPCs).** The metabolism of glucose was assessed by (A) transcriptional levels of genes relevant for glycolysis in ZIKV-infected hi-NPCs relative to non-infected controls. qPCR conducted in four replicates. Dots displaying the values of each patient line. Significance was calculated by two-way ANOVA with Šidák's multiple comparisons post-hoc test. Line graphs displaying (B) the calculated glucose consumption and lactate release from ZIKV-infected and non-infected hi-NPCs, (C) the ratio of change compared to their respective non-infected controls and, (D) comparison of the glycolytic capacity of infected early and late hi-NPCs. *n*=2 viral infections conducted in duplicates of three independent patients' lines. Significance was calculated by mixed-effects model with Holm-Šidák correction. Error bars display mean±s.d. Significance is shown when *P<0.05, **P<0.01, ***P<0.001.

To assess whether ZIKV-induced differential alterations in hi-NPCs were paralleled alterations in the metabolism of glucose, as the main substrate of neuronal progenitors, we quantified the transcripts for key glycolytic genes. Among these genes, HK-1 showed significant increases in ZIKV-infected cells compared to non-infected controls at late stages of ZIKV replication. No significant changes were calculated in the glucose entry receptor GLUT-1 and, analysis of GAPDH showed a significant increased over time exclusively in early hi-NPCs (Fig. 3A). Between cortical progenitors at different stages of maturation, at 48 h.p.i., ZIKV-infected early hi-NPCs showed an increased ratio of 1.42 in the gene expression of HK-1 compared to late hi-NPCs (Fig. 3A). However, at 72 h.p.i., the opposite effect was observed with an increased ratio of 1.26 in ZIKV-infected late compared to early hi-NPCs. At this time-point, ZIKV-infected early hi-NPCs showed significant increased RNA levels of GAPDH at a ratio of 2.64 compared to infected late hi-NPCs (Fig. 3A). In early hi-NPCs, these effects were mirrored at the enzyme level with a significant increase in the lactate release at 48 h.p.i. (*P*=0.045) and 56 h.p.i. (*P*=0.006). In late hi-NPCs, lactate release was not significantly augmented (Fig. 3B). There was no significant increase in the consumption of glucose in neither early nor late hi-NPCs (Fig. 3B). As the stage of differentiation of hi-NPCs reflects the presence of metabolically distinct populations (higher and lower consumers of glucose, Fig. 1J), we examined the effects described above as potential changes in the glycolytic capacity during infection. Significant increases in the glycolytic capacity were exclusively observed in infected early hi-NPCs at 24 h.p.i. (*P*=0.0167), 48 h.p.i. (*P*=0.0022) and 56 h.p.i. (*P*=0.0004) compared to non-infected controls (Fig. 3C) yet, their increased glycolytic rate was not significantly different to that of infected late hi-NPCs (Fig. 3D).

### ZIKV-infected cortical progenitors at different stages of differentiation display specific patterns of mitochondrial alterations during viral replication

**Fig. 4.**
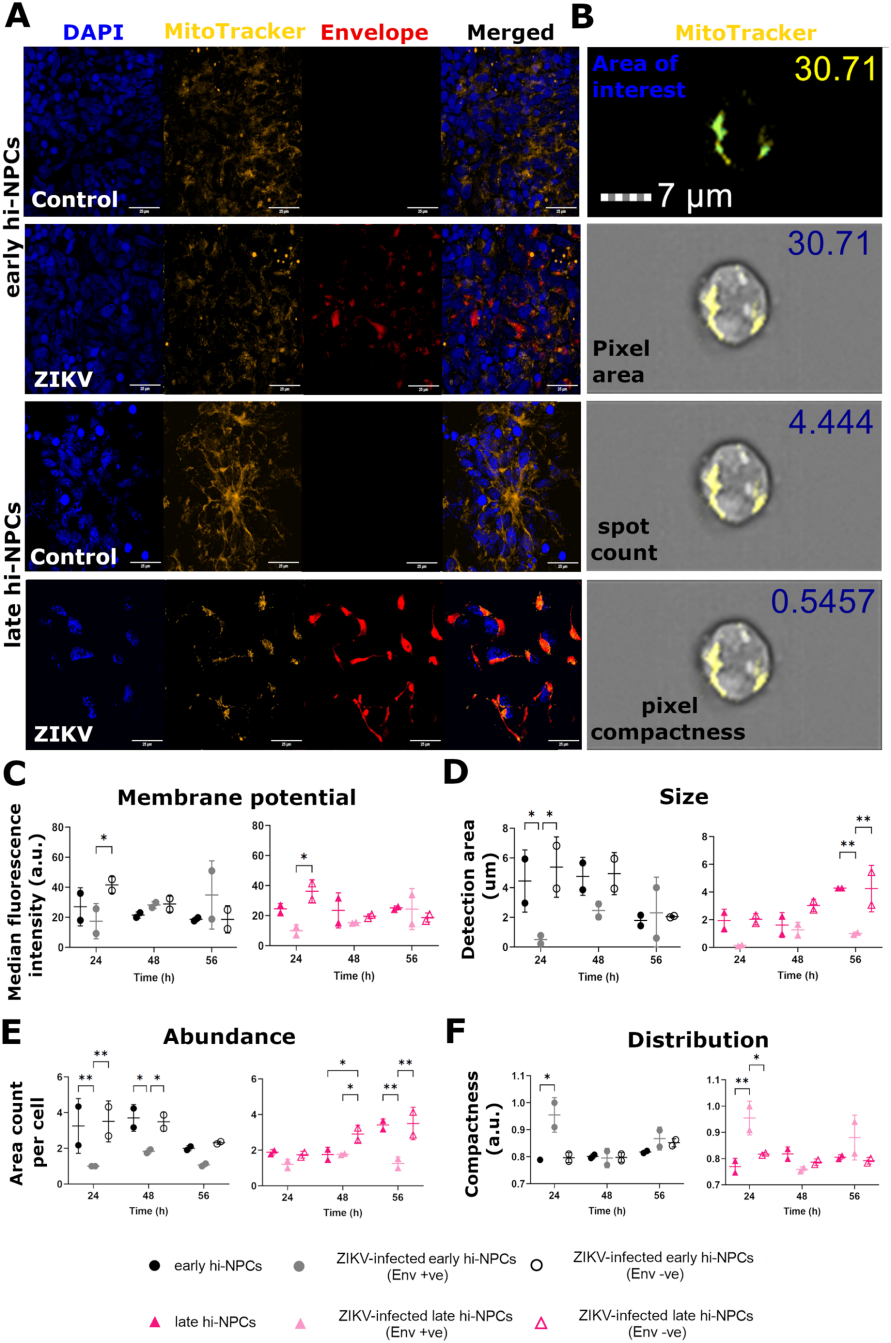
**ZIKV induces neuronal progenitor subtype-specific mitochondrial stress.** Analysis of several characteristics of mitochondria homeostasis in cortical progenitors at different stages of maturation in infected to ZIKV compared to non-infected controls. (A) Representative confocal images with high magnification (60x) of z-stack projections (25 µm) confirming positive staining of mitochondria (orange) and ZIKV Envelope protein (red) in hi-NPCs. DAPI staining (blue). (B) AMNIS Imaging flow cytometer digital images show representative mitochondria staining (yellow) with different quantified parameters, selected by defined mask (blue) in hi-NPCs. Dot plots showing (C) mitochondrial membrane potential by measurements of the median fluorescence intensity of MitoTracker™ Red CMXRos, (D) mitochondria size estimated by the quantification of the total occupied area within the cells, (E) abundance of areas occupied by mitochondria and, (F) distribution of mitochondria determined by the proximity of positive areas within the cells. A minimum of 500 *in focus* cells were analysed per patient line out of 10,000 cells recorded. *n*=1 viral infection conducted in duplicates of two patients' lines. Significance was calculated by mixed-effects model with Tukey's correction. Error bars display mean±s.d. Significance is shown when *P<0.05, **P<0.01, ***P<0.001.

We examined the characteristics of mitochondrial homeostasis by immunofluorescence. We validated the specificity of detection by confocal imaging (Fig. 4A) and analysed ZIKV infected (Env +ve), ZIKV-infected neighbouring cells (Env −ve), and non-infected controls by single cell imaging flow cytometry (Fig. 4B). Distinction between Env +ve and Env −ve was done by the immunodetection of viral proteins within the pool of infected hi-NPCs. Quantification of different mitochondrial parameters using defined mitochondria areas of analysis (Fig. 4B) showed that cellular signalling from Env +ve cells, at 24 h.p.i., induced a significant increase in the mitochondrial membrane potential of Env −ve in early and late hi-NPCs (*P*=0.045 and p=0.0151, respectively) yet, although greater, this increase was not significant compared to non-infected controls (Fig. 4C). We found that mitochondrial alterations in Env +ve hi-NPCs and non-infected controls were distinct during stages of ZIKV replication and that these were specific to each hi-NPC culture. Env +ve early hi-NPCs showed, at 24 h.p.i., a significant reduction in mitochondrial size (*P*=0.0328) compared to non-infected controls (Fig. 4D). These cells also exhibited a significant reduction in mitochondrial abundance at 24 h.p.i. (*P*=0.0091) and 48 h.p.i. (*P*=0.0217) (Fig. 4E). Alterations in Env +ve late hi-NPCs showed that at 56 h.p.i., mitochondrial size (*P*=0.0047) and abundance (*P*=0.002) were significantly reduced compared to non-infected controls (Fig. 4D and E). Lastly, cytoplasmic distribution of mitochondria was significantly reduced at 24 h.p.i. in Env +ve early (*P*=0.0251) and late hi-NPCs (*P*=0.0071) (Fig. 4F).

### ZIKV infection induces specific regulation of lipid metabolic genes depending on the maturation of hi-NPCs

**Fig. 5.**
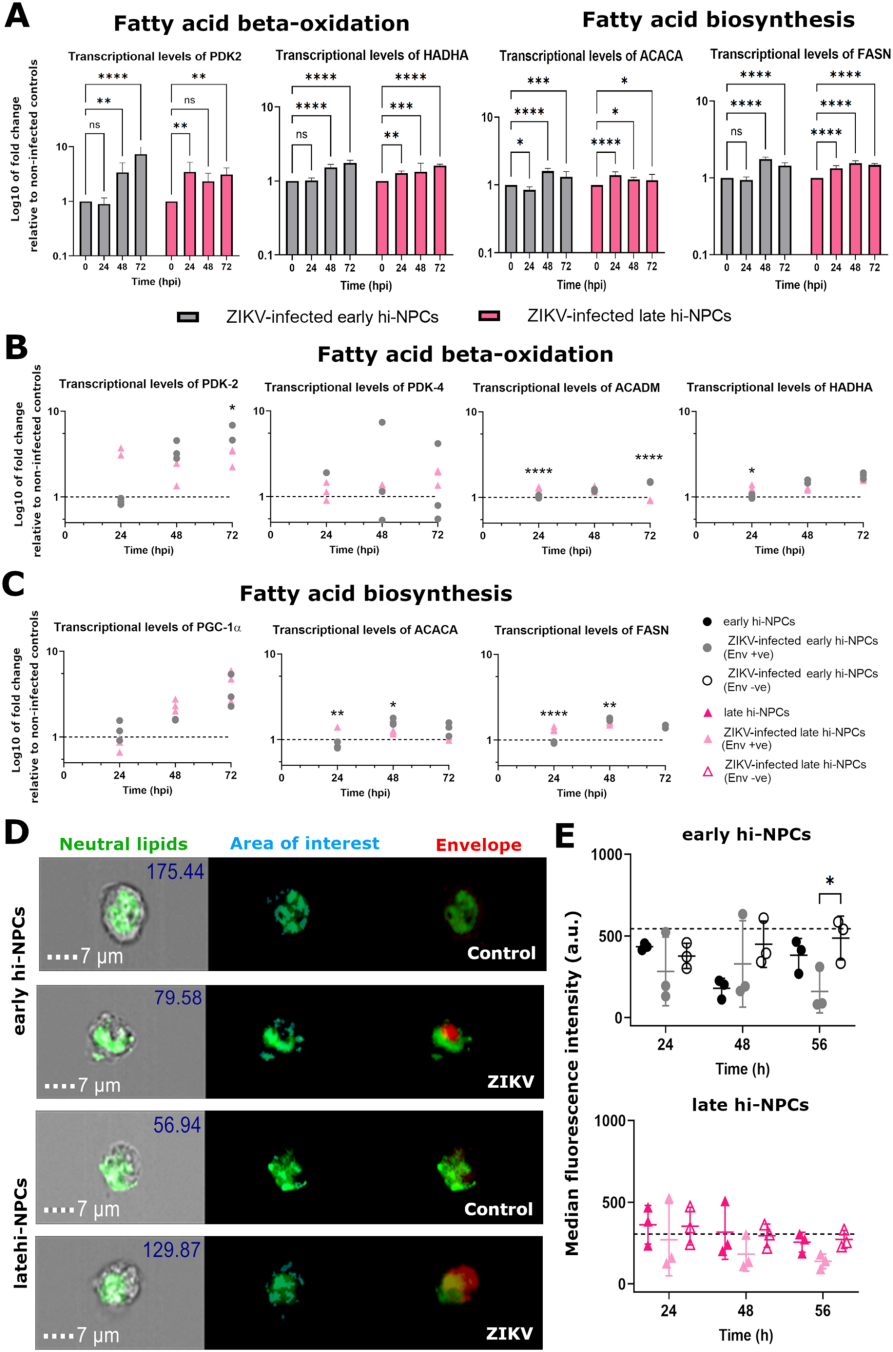
**ZIKV replication induces simultaneous differential gene expression of fatty acid beta-oxidation and lipid biosynthesis in neuronal progenitor cells independently to their maturation.** (A) Bar graphs showing the simultaneous upregulation of genes involved in fatty acid beta-oxidation and biosynthesis in hi-NPCs during ZIKV replication. Bars displaying the results of four independent replicates. Dot plots showing the transcriptional levels of genes relevant in (B) fatty acid beta-oxidation and, (C) fatty acid biosynthesis in ZIKV-infected hi-NPCs relative to non-infected controls. qPCR conducted in four replicates. Dots displaying the values of each patient line. (D) AMNIS Imaging flow cytometer digital images showing representative lipid droplet staining (green), selected by defined mask (light blue), and ZIKV-Envelope staining (red). (E) Dot plots showing the abundance of lipid droplets in ZIKV Envelope positive, Envelope negative, and non-infected hi-NPC controls. Values reflect the median fluorescence intensity (MFI) of CellTracker™ green BODIPY dye. A minimum of 500 *in focus* cells were analysed per patient line out of 10,000 cells recorded. *n*=1 viral infection conducted in three independent patients' lines. Significance was calculated by two-way ANOVA with Šidák's multiple comparisons post-hoc test. Error bars display mean±s.d. Significance is shown when *P<0.05, **P<0.01, ***P<0.001.

The presence of ZIKV likely alters the transcriptional profile of the host cells. We found that ZIKV simultaneously increased the expression of genes involved in of both beta-oxidation and lipid biosynthesis at different time-points during ZIKV replication (Fig. 5A). In addition, gene expression showed that ZIKV replication, particularly at later stages (72 h.p.i.), increased the transcriptional profile but to different rates between early and late hi-NPCs (Fig. 5B and C). PDK2, essential in brain cells to promote fat oxidation (Nakai et al., 2000), showed a significant increase (*P*=0.0153) in early hi-NPCs compared to late hi-NPCs exclusively at 72 h.p.i. (Fig. 5B). The ACADM gene, required for the synthesis of enzymes for the oxidation of medium-chain fatty acids (Grünert et al., 2015), showed at 24 h.p.i. a modest yet significant increase in late compared to early hi-NPCs (late:early RNA ratio of 1.27). In contrast, at 72 h.p.i., early hi-NPCs showed a significantly increased ratio of 1.62 in the levels of ACADM compared to late hi-NPCs (Fig. 5B). A significant increase in the HADHA gene (late:early RNA ratio of 1.26), essential for the synthesis of multi-enzymes within the mitochondrial trifunctional complex (Yang et al., 2022), was also observed exclusively at 24 h.p.i. (Fig. 5B). The analysis of genes involved in lipid biosynthesis (Menendez and Lupu, 2007) showed, only at 24 h.p.i., a significant differential expression of a ratio of 1.64 in ACACA in late compared to early hi-NPCs. In contrast, the level of transcripts of ACACA at 48 h.p.i., showed an increased ratio of 1.33 in early compared to late hi-NPCs (Fig. 5C). Transcriptional levels of FASN, the main biosynthetic enzyme involved in the synthesis of saturated long-chain fatty acids (Menendez and Lupu, 2007), showed similar results at 24 and 48 h.p.i. than those of ACACA. Late hi-NPCs showed a significantly increased ratio of 1.43 compared to early hi-NPCs, exclusively at 24 h.p.i. whilst at 48 h.p.i., we observed significantly opposite results (early:late ratio of 1.12) (Fig. 5C). These results on gene expression were translated at the protein level and followed the kinetics of *de novo* fatty acid synthesis. At 56 h.p.i., in infected early hi-NPCs, when levels of ACACA were greater, FASN showed decreased levels. At this time point, we observed opposite effects in infected late hi-NPCs (Extended Fig. S4). Furthermore, single cell immunofluorescence analysis of lipid droplets in ZIKV-infected hi-NPCs (Fig. 5D) showed that genetic changes were not translated in alterations of the intracellular abundance of neutral lipids (Fig. 5E).

### Initial stages of ZIKV replication dysregulate lipid droplet homeostasis exclusively in early neuronal progenitors

**Fig. 6.**
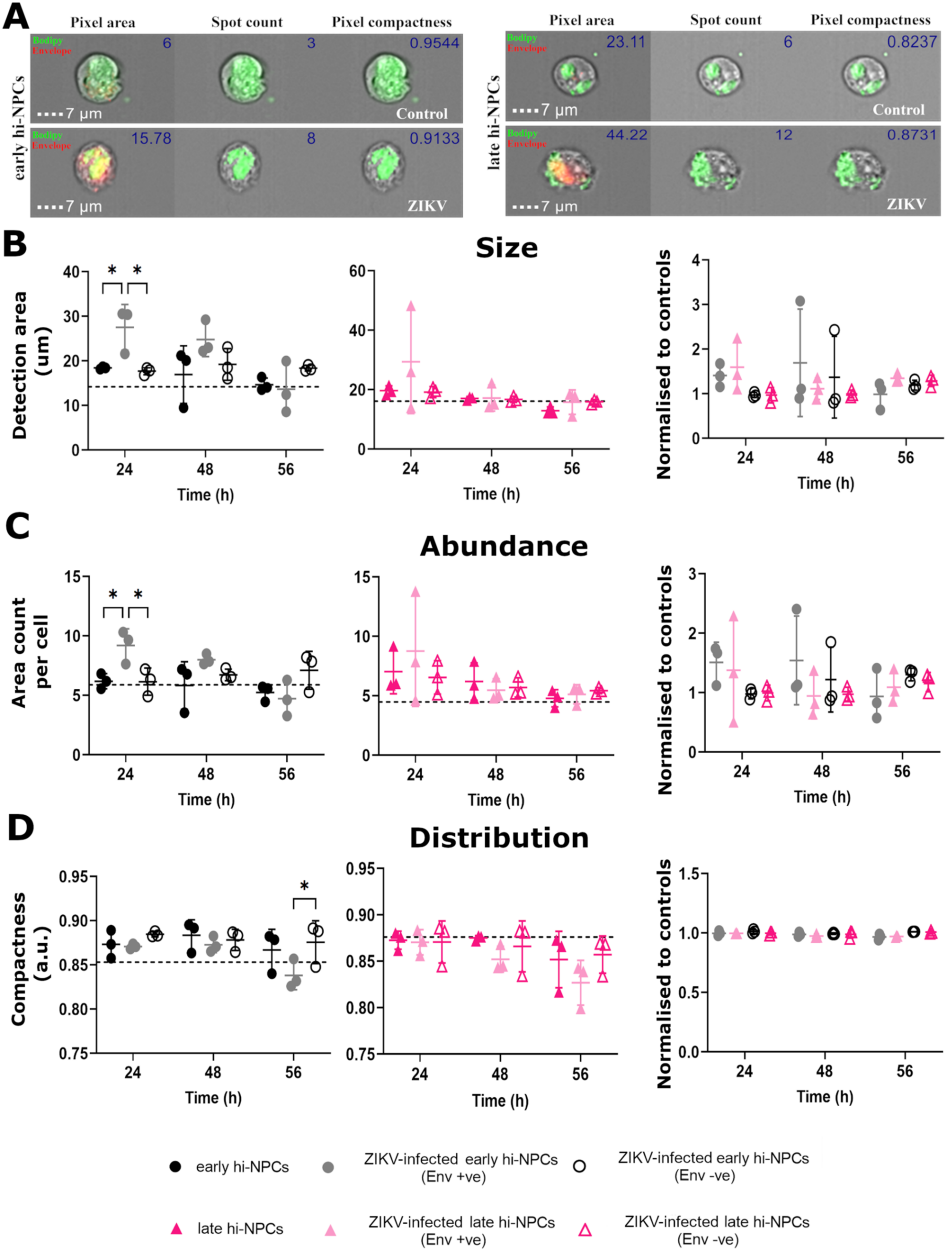
**Early stages of ZIKV replication promotes accumulation of lipid droplets exclusively in less differentiated neuronal progenitor cells.** Analysis of several characteristics of the lipid droplets in hi-NPCs infected to ZIKV compared to non-infected controls. (A) AMNIS Imaging flow cytometer digital images showing representative lipid droplet staining (green), and ZIKV-Envelope staining (red) in non-infected and infected early (left) and late (right) hi-NPCs. Values calculated for the size, abundance and distribution of lipid droplets are represented within the figures. Dot plots showing (B) lipid droplet sizes estimated by the quantification of the total occupied area within the cells, (C) abundance of areas occupied by lipid droplets and, (D) distribution of lipid droplets determined by the proximity of positive areas within the cells. Graphs showing (left) the quantification by hi-NPCs and (right) the ratio of change of each hi-NPC subtype relative to their respective non-infection controls. A minimum of 500 *in focus* cells were analysed per patient line out of 10,000 cells recorded. *n*=1 viral infection conducted in three independent patients' lines. Significance was calculated by two-way ANOVA with Šidák's multiple comparisons post-hoc test. Error bars display mean±s.d. Significance is shown when *P<0.05, **P<0.01, ***P<0.001.

Lipid droplets, important during neurogenesis, are also crucial for the assembly of ZIKV particles (Zhang et al., 2017). Thus, we examined whether in cortical progenitors at different stages of maturation there is a specific alteration of lipid droplet homeostasis during ZIKV replication that may contribute to the disparities in brain damage observed at each trimester. To do this, we studied the biology of lipid droplets in non-infected and infected hi-NPCs by single cell imaging flow cytometry of cells stained for neutral lipids (Figs 5D and 6A). Early Env +ve hi-NPCs exhibited a significantly higher area occupied by lipid droplets (*P*≤0.0242) compared to Env−ve and non-infected control cells, followed by a time-dependent reduction (Fig. 6B). Data corresponding to the abundance of lipid droplets showed similar results with a significant increase (*P*≤0.0189) at 24 h.p.i. in early Env +ve hi-NPCs compared to Env −ve and non-infected controls (Fig. 6C). Late hi-NPCs showed no alterations during ZIKV replication (Fig. 6B and C). Distribution of the area occupied by lipid droplets showed a time-dependent increase in Env +ve hi-NPCs with significant differences at 56 h.p.i. observed exclusively between early Env +ve and Env −ve hi-NPCs (Fig. 6D). These data showed dysregulation of lipid droplets metabolism at early stages of ZIKV replication exclusively in early hi-NPCs yet, we did not find significant differences in the ratio of changes in lipid droplet homeostasis between ZIKV-infected early compared to late hi-NPCs.

## DISCUSSION

3D neurospheres or organoids, differentiated from either fetal or iPSC-derived cells *in vitro* (Nascimento et al., 2019) are used to study fetal brain development. However, these systems show high variability, which complicates the interpretation of differential responses to stimuli, including viral infection (McGrath et al., 2017). Thus, to better understand *in vitro* the effects of ZIKV infection in the metabolism of forebrain progenitors over each trimester, we cultured, using a modified existing 2D protocol (Shi et al., 2012), cortical progenitors obtained from hiPSC (hi-NPC) and characterised the pool of cells produced at two stages of differentiation.

Although the epidemiology of Zika congenital syndrome corresponds to infections with strains of the Asian lineage (Simonin et al., 2017; Aubry et al., 2021; Hamel et al., 2017), phenotypic changes in the viral proteins that may have produced these pathogenic variants mainly correlate with protein mutations in sites involved in the host immune recognition (Hamel et al., 2017; Jung et al., 2022; Sironi et al., 2016). Therefore, these alterations are likely to enhance a greater efficiency of the virus to cross the placental and fetal blood brain barrier yet unlikely to cause differences in the infectivity in 2D and 3D *in vitro* systems (Hamel et al., 2017; Xu et al., 2019; Muffat et al., 2018; Garcez et al., 2016, 2017; Nascimento et al., 2022). To confirm this, we performed a single round of infection in hi-NPCs using a highly pathogenic Asian strain (PRVABC59) and a less pathogenic African strain U-1962 (MP1751) obtaining similar infectivity results (Fig. S5). Considering that *in vitro* passaging, in addition to promote gain of infectivity, decreased cycle time and/or increased ZIKV production (Duggal et al., 2019), may also select for mutations that influence secondary pathways such as cellular metabolism; we extensively studied the potential metabolic disturbances caused by ZIKV in hi-NPCs using the conserved low passaged African strain U-1962 (MP1751). Yet, to avoid investigating processes not relevant for the pathogenesis of ZIKV, we first confirmed that infection with different strains of ZIKV produce similar effects in the metabolism of glucose; the main metabolic pathway during neuronal differentiation (Fig. S5).

The time under culture during the *in vitro* differentiation of hi-NPCs exhibits the presence of morphologically distinct cell populations (Fig. 1B and C). This morphological distinction is observed at two particular timepoints during differentiation in which the pools of hi-NPCs presented similar ratios of undifferentiated neural stem cells and neuroblast cells. However, these pools of hi-NPCs differed in their abundance ratio of radial glial and proliferative progenitors. Further characterisation of these pools showed that the expression of generic cellular markers of neuronal progenitors and proliferation was similar and comparable to that reported by others (Shi et al., 2012; Robbins et al., 2018). Significantly higher expression of Pax6 in late compared to early hi-NPCs may be correlated with Pax6 dynamic expression during neuronal proliferation and differentiation (Hsieh and Yang, 2009). These results on Pax6 correlate to the greater presence of radial glial and proliferative progenitors, which are Pax6 positive cells. Overall, similarities between the detection levels of most of the cellular markers, as well as cell subtypes, suggest morphological differences between the two pools of cortical progenitors may be largely influenced by their stage of maturation due to the time under culture. The different stages of maturation were assessed by metabolic tracing. We showed significant differences in cellular metabolism that reflected the metabolic shift from the cytosolic metabolism of glucose to the mitochondrial oxidation of glucose-derived pyruvate and fatty acids; processes expected to occur upon transition from quiescent to immature neuronal cells (Zheng et al., 2016; Knobloch et al., 2022, 2017; Knobloch and Jessberger, 2017). Early hi-NPC showed high glycolytic flux and low fatty acid oxidation, and late hi-NPCs showed a significantly decreased glycolytic flux and increased rate of fatty acid oxidation. These results, compared to human and mice neuronal progenitors (Zheng et al., 2016; Knobloch et al., 2022, 2017; Knobloch and Jessberger, 2017), suggest that early hi-NPCs are likely to contain within the pool of cells, cells with features of quiescent radial-glia like cells and self-renewal progenitors. In contrast, the metabolic profile of late hi-NPCs represents populations of proliferative progenitors and immature brain cells.

These results were supported by the long-term estimation of the glycolytic capacity that, although it did not differ between hi-NPCs, it showed early hi-NPCs consumed higher levels of glucose, potentially due to differences in the length of their cell cycle. Thus, early and late hi-NPCs may recapitulate different stages of forebrain development by the presence of metabolically distinct populations of brain cortical progenitors which their abundance changes over each trimester (Gilbert-Jaramillo et al., 2019; Rothan et al., 2019).

Immune responses in cortical progenitors remains unclear (Lin et al., 2019a) with data suggesting that more differentiated neuronal cells possess stronger interferon responses compared to undifferentiated neural stem cells (Castorena et al., 2008; Farmer et al., 2013). ZIKV is a known antagonist of interferon-I signalling (Wu et al., 2017; Hu et al., 2019; Kumar et al., 2016), therefore, one would expect a greater ZIKV replication in early hi-NPCs. However, we showed that late hi-NPCs accumulate more ZIKV transcripts that result in a significantly greater release of virions (Fig. 2C and F). Thus, lower levels of ZIKV replication in early compared to late hi-NPCs may be correlated with the significantly greater cell death and therefore reduction of host cells to act as viral reservoirs. This hypothesis, however, does not apply for decay in ZIKV production in late hi-NPCs at later stages of replication (Fig. 2F). Thus, a plausible explanation for the decrease in the kinetics of ZIKV release in late hi-NPCs after 56 h.p.i. may involve an increase in the native immune responses due to maturation of hi-NPCs by their time under culture yet, further testing is needed.

Intracellular translation of viral RNA proteins causes several responses that may differentially contribute to the cell-type specific pathogenesis. Thus, no significant differences in the accumulation of intracellular NS1 and envelope protein (Env) between hi-NPCs (Fig. 2D and E) may suggest that different outcomes of ZIKV infection between infected early and late hi-NPCs may be influenced by their maturation state. ZIKV infection in neuronal progenitors is known for causing cell death (Devhare et al., 2017; Li et al., 2016a,b). However, we observed significant cell death exclusively in infected early hi-NPCs compared to controls at later stages of ZIKV replication. These findings may translate to the fetal pathogenesis of ZIKV in which detrimental alterations in brain formation are mainly observed when infection occurs during the first trimester of pregnancy (el Costa et al., 2016; Kleber de Oliveira et al., 2016). Interestingly, a significant increase in proliferation of early hi-NPCs at early stages of ZIKV replication correlates with data from Souza et al. (2016), which also displays a greater cell number at initial timepoints during ZIKV replication with subsequent significant cell death (Souza et al., 2016).

Nuclear disruption in neuronal progenitors constitutes a feature of ZIKV replication, yet this is the first report showing differential changes in nuclear morphology between pools of cortical progenitors containing cells at different stages of maturation. Our main finding was the vast presence of viral perinuclear replication centres (Caldas et al., 2020) in late hi-NPCs but not in early hi-NPCs (white arrows, Fig. S3 and Fig. S6). Although not significant, potentially due to nuclear effects been limited to virus-containing infected cells, modest reduction in nuclear size and increased nuclear circularity in early hi-NPCs at specific time-points may be related to cellular shrinkage and death at the time of analysis (Anfasa et al., 2019).

Hijacking of cellular metabolism by flaviviruses is a distinct process that varies between virus strains and host cells. For example, Dengue virus (DENV) infection directly increments and requires lactate glycolysis for its replication and, whilst this is not observed in West Nile virus (WNV), lipid biosynthesis is incremented during infection of both viruses; DENV significantly augments fatty acid biosynthesis and WNV cholesterol biosynthesis (Gilbert-Jaramillo et al., 2019; Rothan et al., 2019). Therefore, understanding whether ZIKV infection causes specific metabolic changes among cortical progenitors at different stages of maturation or if it recapitulates metabolic features observed in other flaviviruses, potentially highlight mechanisms that can be targeted for therapeutics to prevent severe consequences to the developing fetal brain.

ZIKV infection resulted in transcriptional upregulation of genes involved in glycolysis in hi-NPC. However, our analysis on the transcriptional upregulation of genes involved in glycolysis showed that the gene codifying for the main glucose entry receptor in brain cells (GLUT-1) did not vary in either pool of hi-NPCs (Fig. 3A). This potentially as our cells within both cultures already at their maximum glucose uptake capacity due to being cultured in hyperglycaemic conditions.

Increased glucose consumption during ZIKV replication has been previously reported using a diversity of human and non-human cell lines (Thaker et al., 2019; Yau et al., 2021; Singh et al., 2020; Lin et al., 2019b), yet we exclusively observed this phenomenon in early hi-NPCs. This may suggest that ZIKV infection requires glycolytic by-products that in less mature and highly glycolytic cortical progenitors (early hi-NPCs) may be rapidly utilised for cellular homeostasis. Another possibility is that ZIKV replication in highly glycolytic cells increments glycolysis to support additional processes such as redox clearance and nucleotide production (McGrath et al., 2017), and/or to signal cell survival-related pathways necessary for efficient viral replication (Pang et al., 2021; Tiwari et al., 2020).

The role of mitochondria in the orchestration of metabolic pathways is likely to be manipulated by ZIKV (Li et al., 2021). We showed that ZIKV infection causes time-dependent dysregulation of mitochondrial homeostasis with differential effects between early and late hi-NPCs. The dysregulations could be due to ZIKV differential hijacking of the available intracellular pools of metabolites in early and late hi-NPCs. The difference in intracellular metabolite pools may be due to a lower percentage of mitochondrial oxidative cells in early hi-NPCs compared to late hi-NPCs in which most cells rely on mitochondrial oxidation of fatty acid and glucose. In Env +ve early hi-NPCs, we observed mitochondrial dysregulation in the form of reduced size and abundance when compared to Env −ve and non-infected controls during early stages of viral replication (24 h.p.i. and 48 h.p.i.). This may suggest that ZIKV infection increases mitochondrial fragmentation and mitophagy (Scott and Youle, 2010; Zorov et al., 2019). These processes are known to impact cellular respiration by the decrease of the aerobic capacity in skeletal and cardiac muscle cells (Philp et al., 2021; Gu et al., 2021). Potentially recapitulated in the brain, these processes aligned with our results on increased lactate production (Fig. 3B).

Reduced mitochondrial membrane potential was observed in both Env +ve early and late hi-NPCs at 24 h.p.i. when compared to Env −ve cells but not to non-infected controls. These findings are different to those in ZIKV-infected human neuronal stem cells, human retinal and human hepatoma cells (García et al., 2020) in which ZIKV-infected cells had higher mitochondrial membrane potential. In a similar fashion, reduced distribution of mitochondria was found in both Env +ve hi-NPCs at 24 h.p.i. when compared to Env −ve and non-infected controls. Overall, the effects on mitochondrial dysregulation observed at 24 h.p.i. are of high relevance during the kinetics of ZIKV replication as these are directly influenced by simultaneously infected cells (Env +ve) upon inoculation. At later time-points (48 h.p.i. and 72 h.p.i.), the observed effects are influenced by a mixture of previously and newly infected cells, thus, results may be subjected to a greater variability and/or diminished by the kinetics of infection potentially masking the mechanisms displayed at 24 h.p.i.

Altogether, these results showed for the first time that ZIKV-induced dysregulation of mitochondrial homeostasis differs between cortical progenitors at different stages of maturation and that those mitochondrial changes, at specific time-points during viral replication, are exclusively observed in infected and not in neighbouring cells. Thus, highlighting the importance of time-course mitochondrial tracing to better understand the role of energy metabolism in the pathogenesis of ZIKV.

Results from human monocytes and *Drosophila* suggest ZIKV infection increases beta-oxidation of fatty acids at early time-points (Tiwari et al., 2017; Harsh et al., 2020). However, results obtained from mouse models of ZIKV pathology showing decreases in the TCA cycle, oxidative phosphorylation, and cytosolic levels of NAD^+^ (Yau et al., 2021; Pang et al., 2021) may suggest a decreased beta-oxidation of fatty acids. We showed that ZIKV simultaneously increases the expression of genes involved in fatty acid beta-oxidation and biosynthesis yet to different rates between early and late hi-NPCs. The rate of increase of gene expression of fatty acid beta-oxidation showed significantly greater alterations in late hi-NPCs at early timepoints during infection that then transitioned to be greater in early hi-NPCs at later timepoints. The opposite pattern was observed for the screened genes involved in fatty acid biosynthesis. Thus, our findings may enable to understand the current disparities in the literature regarding ZIKV infection and metabolic dysregulation (Yau et al., 2021; Pang et al., 2021; Tiwari et al., 2017; Harsh et al., 2020) as we showed that complex alterations in the transcriptional levels of metabolic genes during ZIKV infection may be specific to the stage of differentiation of brain cells.

Lipid droplets are multifunctional organelles that comprise aggregates of several types of lipids that can be exploited by ZIKV to support its replication (Zhang et al., 2017) yet their entire role during ZIKV infection remains unclear. Changes in lipid droplet homeostasis during the kinetics of ZIKV infection have been showed in less relevant non-neuronal models (Zhang et al., 2018). Thus, we examined the homeostasis of lipid droplets by their number and size during ZIKV infection. Our results showed that differential effects in the lipid droplet content between hi-NPCs were restricted to ZIKV Env +ve early hi-NPCs. The significant increase in the lipid droplet abundance in Env +ve compared with Env −ve cells at 24 h.p.i. are in contradiction with the findings from a study of a similar kind in placental stromal cells that showed significant increases in lipid droplets in Env −ve cells (Chen et al., 2020). This, potentially reflecting differences in the cellular response to ZIKV infection between cell types and/or the dynamics of lipid droplet utilization during sustained ZIKV infection.

### Conclusion

**Fig. 7.**
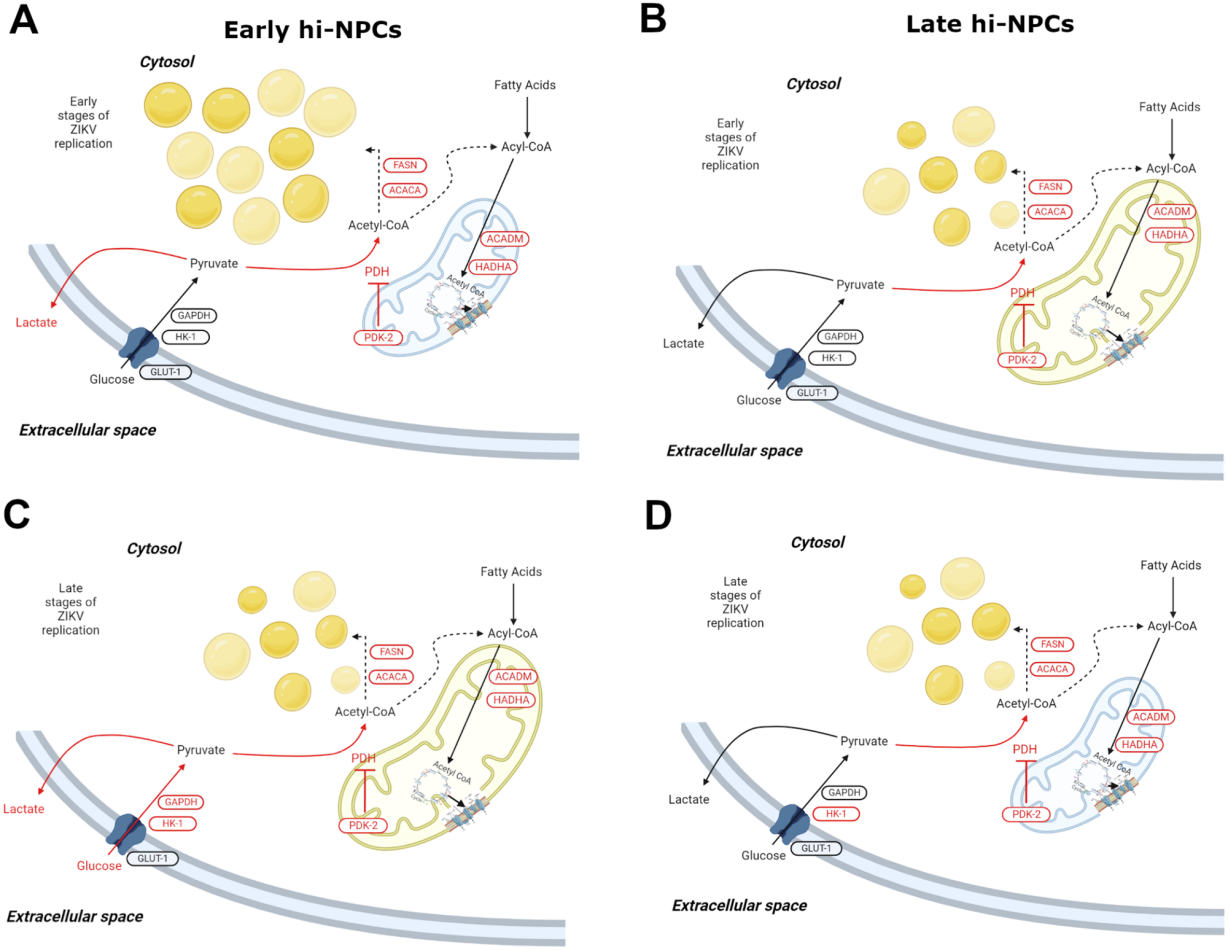
**ZIKV-induced neuronal progenitor subtype-specific metabolic alterations.** Schematic representation of the differential metabolic dysregulation caused by ZIKV replication in cortical progenitors at different stages of maturation. (A) Increased abundance and size of lipid droplets with reduced mitochondrial size and abundance (blue colour) in Env +ve early hi-NPCs during early stages of ZIKV replication. (B) Normal abundance of lipid droplets, normal mitochondria size and abundance (yellow colour) in Env +ve late hi-NPCs during early stages of ZIKV replication. (C) Normal abundance of lipid droplets, normal mitochondria size and abundance (yellow colour) in Env +ve early hi-NPCs during late stages of ZIKV replication. (D) Normal abundance of lipid droplets with reduced mitochondrial size and abundance (blue colour) in Env +ve late hi-NPCs during late stages of ZIKV replication. Black arrows representing normal fluxes of each main metabolic pathway. Black dotted arrows representing potentially dysregulated metabolic pathways. Red arrows highlighting increased pathways assessed by protein expression. Gene/protein names in red corresponding to increased levels whilst those in black corresponding to unaltered levels compared to non-infected controls.

To conclude, we showed that cortical progenitors of the forebrain differentiated from hiPSC (hi-NPCs) can be distinguished into different maturation stages *in vitro* by their metabolic profile of utilisation of glucose and fatty acids. These pools of cortical progenitors showed similar ratios of fetal brain cells during differentiation thus, their characteristic metabolic profile suggest a transition in maturation that may resemble the fetal brain subpopulations over each trimester. We found that the metabolic differences of these hi-NPCs are distinctively hijacked by ZIKV to support sustained replication. ZIKV replication induced an increase in the glycolytic capacity of early hi-NPCs (Fig. 3). Mitochondrial dysregulation was also observed in hi-NPCs yet distinct to their maturation and stage of ZIKV replication (Fig. 4) whilst stress on lipid droplet homeostasis was exclusive to early hi-NPCs at early stages of ZIKV replication (Fig. 7). Intrauterine growth restriction and neurodevelopmental disorders such as epilepsy, schizophrenia and autism comprise alterations in the metabolism of glucose and mitochondrial oxidation (Devaskar and Chu, 2016; Jones et al., 2020; Vannucci and Vannucci, 2000; Jha et al., 2016; A et al., 2021) with impairment in neuronal communication but not significant cell death. These alterations may well correlate with some processes observed in infected late hi-NPCs, thus, potentially explaining the abnormalities observed in newborns when ZIKV infection occurs at later stages during pregnancy. Of relevance, the lack of glucose receptors in the fetal brain (GLUT1 deficiency syndrome) (Gilbert-Jaramillo et al., 2019; Blonz, 2016) causes congenital microcephaly and fetal neurodevelopmental abnormalities; shared phenotypes with Zika congenital syndrome (Blonz, 2016; Wen et al., 2017; Merfeld et al., 2017; Mlakar et al., 2016). Although we observed an upregulation of glycolysis during ZIKV infection, restriction of cortical progenitors from glycolytic intermediates spared for ZIKV replication may mirror the intracellular stress of GLUT1 deficiency syndrome. This may explain the phenotypes observed in newborns when infection occurs during the first trimester where the pool of brain progenitors is expected to comprise immature cells like our early hi-NPCs. Thus, our results may suggest that the distinct ZIKV-induced fetal brain damage over each trimester correlates to specific metabolic alterations in infected brain cells subjected to their maturation stage.

## MATERIALS AND METHODS

### Propagation and titration of ZIKV stocks

ZIKV strains [MP1751 Virus Pathogen Database and Analysis Resource (ViPR) - Flaviviridae - Flavivirus Zika virus Strain MP1751, Virus Collection: 1308258v Zika virus, and PRVABC59] were propagated on Vero cells (ATCC CCL-81) passages 56 to 62. Cells were firstly incubated with the viral inoculum for 1.3 h at room temperature (RT) followed by further four days at 37°C, 5% carbon dioxide until supernatant was collected upon evident cytopathic effect (CPE). Virus titration conducted by plaque assay was done by serial 10^−1^ viral dilutions in Vero cells at a density of 2.5×10^5^ cells. 1.5% carboxymethyl cellulose overlay was added after 2 h incubation to prevent viral spread. Plates were further incubated for 80 h under these conditions before plaques were reveal by the staining using Amido black for 30 min at RT. Plates were imaged using a Molecular Imaging ChemiDoc™ XRS+. Plaque forming units (p.f.u.) were calculated as follows: *p*.*f*.*u*.=(*n*/0.1)/*D* where *n*=average number of plaques, 0.1 is the volume of virus added in ml, and *D*=dilution factor.

### Viral thermostability

Thermostability of ZIKV (strain MP1751) at 37°C was assessed by plaque assay. 500 μl of media-containing virus were added to wells of a 24-well plate, each corresponding to a specific time point. 350 μl of media were collected and frozen down at −80°C before titration.

### ZIKV infection in 2D cultures of cortical progenitors (hi-NPCs)

For viral infection in hi-NPCs, cells were incubated for 2 h at 37°C, 5% carbon dioxide with media-containing ZIKV at an M.O.I. of 1 (calculated by the cell number post-plating due to differential cell survival/replication post-thawing between early and late hi-NPCs). Media-containing virus was then removed, and cells were washed once with PBS before adding fresh neuronal maintenance medium (NMM) and incubated under the same conditions. Mock infections were done by exposing cells to supernatants of Vero cells cultured for 96 h.

### Culture of Vero CCL-81 cells

Vero CCL-81 cells passaged every 2-3 days were cultured at 37°C, 5% carbon dioxide in DMEM high glucose supplemented with 10% FBS. Cell passage was done at ∼90% confluency. All cells were pelleted by centrifugation at 400 ***g*** for 5 min at 4°C prior to resuspension. For the preparation for plaque assays, media from Vero cells was replaced for DMEM high glucose supplemented with 1% FBS and 1% Penicillin-Streptomycin (P/S).

### hiPSCs

The hiPSC lines used in this study have been reported elsewhere: SFC840-03-03 (Fernandes et al., 2016), SFC841-03-01 (Beers et al., 2012) and, SFC856-03-04 (Haenseler et al., 2017). Patient lines were derived from dermal fibroblasts from disease-free donors recruited through StemBANCC (Morrison et al., 2015) and the Oxford Parkinson's Disease Centre: participants were recruited to this study having given signed informed consent, which included derivation of hiPSC lines from skin biopsies [Ethics Committee: National Health Service, Health Research Authority, NRES Committee South Central, Berkshire, UK, who specifically approved this part of the study (REC 10/H0505/71)]. Non-sendai reprogramming (Cytotune, Life Technologies) was used to reprogram fibroblast cells into hiPSCs. hiPSCs were cultured in defined, open-source medium termed OXE8 (Vaughan-Jackson et al., 2021). Cells, resuspended as clumps by using 0.5 mM EDTA, were plated onto Geltrex^TM^ precoated plates and cultured at 37°C, 5% carbon dioxide. Media changes were done every 24 h using not supplemented OXE8 (Vaughan-Jackson et al., 2021). At ∼90% confluency, cells were passaged. Cells were passaged every 2-3 days and, after passage four, cells were either differentiated or stored in liquid nitrogen (LN_2_).

### Generation and culture of cortical progenitor cells (hi-NPCs)

hiPSC passaged two to four times post-thawing at a confluency of ∼95% were induced to neuronal lineages using a modification of a protocol reported elsewhere (Robbins et al., 2018). For neuronal differentiation, 6-7 days incubation in neuronal induction media [NIM: NMM, 100 mM LDN193189 (SML0559), 10 µM SB431542 (ZRD-SB-50)] were required. Media was replaced daily and cells were observed under the microscope to assess the formation of a neuroepithelium-like sheet. If detachment (normally occurring after day 6), cells were washed once with PBS and incubated with 0.5 mM EDTA in PBS for 5 min at 37°C, 5% carbon dioxide. Cells were pelleted at 300 rcf for 3 min and resuspended, as clumps, in neuronal maintenance media [NMM: 50% Neurobasal medium, 50% DMEMF:F12 medium, 2 mM Glutamax, 1X B-27 Supplement (17504-044), 1X N-2 Supplement (17502-048)] supplemented with 10 µM ROCKi. Media was replaced daily with NMM. After 5 days, cells were replated (split 1:2) as detailed before. Following three extra days in culture, cells were either replated (split 1:2) or stored in LN_2_ (early hi-NPCs). Two additional passages, each of 3 days, were further conducted. Cells from the final passage were replated as single cells suspension (late hi-NPCs). For long term storage in LN_2_, cells were resuspended in freezing media composed of NMM supplemented with 10% DMSO and 5 µM ROCKi.

### Radioactive metabolic tracing (Board et al., 2017; Collins et al., 1998; Malandraki-Miller et al., 2019)

Radioactive metabolic tracing of cytosolic glucose utilisation and, mitochondrial oxidation of glucose and fatty acid were assessed in one of the patient lines (SFC841-03-01) used in this study. The metabolic assessment was done to differentiate the favoured metabolic pathway of each pool of cortical progenitors at the two different times under culture as a mean to estimate the maturation of each of the pools. Due to safety regulations, this assessment was exclusively done in non-infected hi-NPCs. As a readout of metabolic disturbances upon ZIKV infection, protocols to measure glucose consumption and lactate release from the culture medium were used. Each radioactive tracing is detailed below:

### Glycolytic flux

hi-NPCs pre-plated for 10 h (5 h post-attachment) were fed with culture medium containing 5-^3^H-glucose {Culture media was composed of no glucose DMEM supplemented with 5 mM glucose and 0.2 µCi/ml or 0.00074 MBq/ml of glucose, D-[5-^3^H(N)] (PerkinElmer)} and incubated at 37°C, 5% carbon dioxide for 4 and 6 h after which cell supernatants from duplicate plates were collected at each respective time-point and stored at −20°C. 5-^3^H-glucose was converted via glycolysis into fructose-6-phosphate releasing ^3^H_2_O into the culture medium. Released ^3^H_2_O requires separation from unconverted 5-^3^H-glucose. This separation process was done using the ion-exchange chromatography separation (Dowex) method. Dowex solution was prepared by mixing 250 g AmberChrom^®^ 1X4 chloride form, 100-200 mesh with 1.25 M NaOH and 1.61 M boric acid. The mixture was mixed gently and repeatedly washed with dH_2_O until pH 7.5 was reached. Dowex solution was added to glass Pasteur pipettes (VWR 612-1701, Avantor; 612-1701) containing glass wool. After this, 200 μl of the media were added to the Dowex column and incubated for 15 min allowing ^3^H glucose to bind to the column. ^3^H_2_O was eluted to the vials by rinsing them twice with dH_2_O. For data normalisation, radioactivity was measured using Tri-Carb 2800TR Liquid Scintillation Analyzer (Perkin Elmer). 0.2 ml medium at time point 0 was used as a control to determine the specific activity of the buffer.

### Glucose oxidation

Glucose oxidation was measured using a modified protocol (Board et al., 2017) based on the original CO_2_ capture method by Collins et al. (1998). hi-NPCs pre-plated for 10 h in a 24-well plate were washed once with PBS and fed for 2 h and 4 h with no glucose DMEM supplemented with 12 mM glucose containing 0.2 μCi/ml [D-[14C(U) glucose: 1mCi-37MBq]. To measure CO_2_ as an estimation of glucose oxidation cells were killed by incubation with perchloric acid for 1 h post-feeding allowing CO2 to be released. The released ^14^CO_2_ was trapped in KOH-soaked filter papers. These papers were analysed using a scintillation counter. For data normalisation, 0.01 ml of medium at time point 0 h was used as a control to determine the specific activity of the buffer.

### Oleic acid oxidation

Radioactive tracing of oleic acid oxidation was based on the detection of ^3^H_2_O produced from ^3^H oleate in the electron transport chain. hi-NPCs pre-plated for 10 h were fed for 4 and 6 h at 37°C, 5% carbon dioxide with no glucose DMEM, 2% BSA, 0.3 mM oleate and 0.2 μCi/ml of oleate, [9,10-3H(N): 1mCi-37MBq] (PerkinElmer). For media preparations, oleate was heated to melt them down and was simultaneously added with ^3^H oleate to the BSA to ensure the same binding ratio. Folch extraction method, modified and reported by Malandraki-Miller et al. (2019), was used to separate the ^3^H_2_O from the ^3^H oleate. 0.5 ml of perfusate was pipetted in 15 ml falcon tube containing: 1.88 ml of chloroform:methanol (1:2 v/v) solution, 625 μl chloroform and 625 μl KCL-HCl solution (2 M KCl, 0.4 M HCl). The solution was then rotated on a laboratory stuart rotator SB3 at 40 rpm for 1 h. After rotation, top aqueous layer was collected, and bottom organic layer was discarded. The aqueous layer was then exposed to 1 ml chloroform, 1 ml methanol and 0.9 ml KCL-HCL and rotated for an extra 1 h at 40 rpm. 0.5 ml the top aqueous layer was used to count the radioactivity using a Tri-Carb 2800TR Liquid Scintillation Analyzer (Perkin Elmer). For data normalisation, 0.5 ml of medium at time point 0 was used as a control to determine the specific activity of the buffer.

### Confocal microscopy

For detection of neuronal markers, permeabilised fixed cells were incubated overnight with primary antibodies in PBS with 0.3% triton X-100 and 5% DS at 4°C. Antibodies dilutions were as follow: anti-Pax6 (1:200, BioLegend; Poly19013), anti-Pax6 (1:200, GeneTex; GTX113241), anti-Tbr2 (1:50, Abcam; Ab23345), anti-Tuj1 (1:1000, BioLegend; 801202), anti-Sox2 (1:150, Merck; Ab5603), anti-Iba1 (1:500, Abcam; Ab5076), anti-MAP2 (1:100, Sigma-Aldrich; M4403), anti-Nestin (1:100, Abcam; Ab93666), anti-NeuN (1:150, Merck; MAB377) and, anti-pH-3 (1:50, Abcam; ab14955). Incubation with secondary antibodies [1:500; goat anti-mouse and goat anti-rabbit Alexa Fluor 488, Alexa Fluor 546, Alexa Fluor 555, Alexa Fluor 647 and, Alexa Fluor 750, or donkey anti-goat Alexa Fluor 488 and Alexa Fluor 647 (all from Invitrogen)] was conducted for 2 h at RT. Cells were incubated for 5 min with 1:10,000 dilution of DAPI and mounted on a glass slide. For staining of mitochondrial membrane potential, live hi-NPCs were incubated with 130 nM MitoTracker™ Red CMXRos for 30 min in the dark at 37°C, 5% carbon dioxide before fixing with 2% PFA for 30 min. Cells were permeabilised and blocked in FACs buffer (PBS supplemented with 1% FBS, 10 µg/ml human-IgG, and 0.01% Sodium azide) 0.01% saponin for 30 min at RT. Cells were incubated with anti-ZIKV Envelope (1:50, GeneTex; GTX133314) overnight at 4°C. Lipid droplet staining (1:5000 BODIPY™ 493/503) was then conducted for 1 h at RT together with 1:2000 DAPI and goat anti-rabbit Alexa Fluor 647. Cells were then mounted, and images were acquired with a Zeiss LSM710 confocal microscope. Acquisitions were performed in five random fields captured under 63x magnification, 1.2 zoom of six sections per image, each section of 5 µm. Images were processed and/or quantified using Fiji-ImageJ2 version 2.3.0/1.53f.

### Flow cytometry

Resuspended hi-NPCs were live stained (LIVE/DEAD™ Fixable Violet Dead Cell Stain Kit) following the manufacturer's procedure. Cells were then fixed with 2% PFA in PBS for 15 min or live stained with markers of brain cell types prior to fixation. Staining of fixed cells was conducted by an initial permeabilization (0.3% saponin FACs) for 1 h at RT followed by an overnight incubation at 4°C incubation in primary antibody solutions Primary antibodies: anti-Pax6 (1:150, GeneTex; GTX113241), anti-Tbr2 (1:100, Abcam; Ab23345), anti-Sox2 (1:70, Merck; Ab5603), anti-MAP2 (1:100, Sigma-Aldrich; M4403), anti-Nestin (1:70, Abcam; Ab93666), anti-NeuN (1:200, Merck; MAB377), anti-Ki67 (1:500, Abcam; ab16667) and anti-S100B (1:30, Sigma-Aldrich; S2532). Secondary staining consisted of 1:2000 goat anti-rabbit Alexa Fluor 555 and goat anti-mouse Alexa Fluor 647, both from Invitrogen. Live staining of markers of brain cell types was conducted following the manufacturer's procedure (BD Biosciences Human Neural Cell Sorting Kit). Shortly, after live/dead staining, cells were filtered through a 70 µm cell strainer and resuspended in 5 mM EDTA. Cells were then stained with either primary conjugated antibodies or isotype controls for 30 min at 4°C. Finally, cells were washed once and resuspended in 1% PFA in FACs buffer for 30 min. Data acquisition was done with Cytoflex LX (Beckman Coulter) CytExpert software. Data analysis was performed using FlowJo V10.8.1.

### Fluorescence imaging

For the analysis of nuclear changes during ZIKV infection, hi-NPCs plated on 96-well plates were fixed with 2% PFA for 15 min at RT and stained with 1:10,000 DAPI dilution in PBS for 10 min. Cells were imaged with an EVOSFL Auto. Nuclear size and shape, of at least 300 nuclei per condition, were calculated using Fiji-ImageJ2 version 2.3.0/1.53f.

### Imaging flow cytometry (Imagestream^®^)

Resuspended hi-NPCs were live stained for mitochondrial membrane potential, fixed and permeabilised as previously detailed. Overnight incubation at 4°C with primary antibody (FACs buffer 0.1% saponin, 1:100 anti-NS4A (GeneTex; GTX133704) and 1:100 anti-NS1 (Abcam; ab214337) was conducted prior to secondary staining with 1:1000 goat anti-mouse Alexa Fluor 546 and goat anti-rabbit Alexa Fluor 750. Cells were then re-stained over 3 h at RT with FACs buffer 0.1% saponin, 1:100 anti-Envelope. Secondary goat anti-rabbit Alexa Fluor 647 staining was conducted in parallel with 1:10,000 Bodipy™ staining for 1 h at RT. 1:10,000 DAPI staining was then carried out for 5 min. Cells were kept in PBS and data acquisition was done with an Amnis^®^ ImageStream^®X^ MkII (Luminex) Inspire 10 software. Data analysis was performed using IDEAS software V6.2.

### Extracellular glucose measurements

The concentration of glucose in the culture media was detected using a miniaturization of a commercial protocol (Merck; GAHK20). 100 µl of culture media were diluted 1:10 and 1:20 in dH_2_O. 20 µl of each dilution were added per well on a F-bottom 96-well plate. Glucose solution was freshly made up and 100 μl were added onto each sample. Plates were incubated at 35°C for 15 min inside a SpectraMax M5 plate reader. NADH absorbance was read at 340 nm wavelength. Glucose values from the samples were calculated by curve fitting to a known standard curve.

### Extracellular lactate measurements

Lactate from the culture media was calculated based on the NADH absorbance at 340 nm wavelength. 5 µl of sample were added per well and incubated with 100 µl of freshly made running buffer solution (3.5 ml Glycine buffer pH 9.2 (per 100 ml: 4.5 g of glycine 99%, 1.3 ml hydrazine hydrate, 95 ml dH2O), 6.5 ml dH2O, 165 μl Bovine lactate dehydrogenase 1 K units, 10 mg NAD^+^). for 20 min at 37°C inside a SpectraMax M5 plate reader. NADH absorbance was read at 340 nm wavelength. Lactate values were calculated by curve fitting to a known standard curve using sodium L-lactate.

### Cell survival measurements

Cell survival was determined by the quantification of the lactate dehydrogenase (LDH) levels released from plated cells after induced death. At every time point of assessment, culture medium from cells was removed and replaced by culture medium containing 1% triton-X 100. Cells were incubated for 30 min at 37°C, 5% carbon dioxide after which cells were flushed using a micropipette. Medium was collected, spun at 10,000 ***g*** for 5 min to discard cell debris and, 90 µl of supernatants were collected and kept at 4°C until measurement. 20 µl of supernatants were incubated with 100 µl of reaction buffer (3.5 ml Glycine buffer pH 9.2), 6.5 ml dH2O, 10 mg NAD^+^, 40 mM L-sodium lactate) for 30 min at 37°C. NADH absorbance equivalent of the amount of lactate converted by the LDH released from cells was read at 340 nm wavelength. LDH values were transformed to cell number by curve fitting to a known standard curve generated using specific cell numbers. For experiments conducted in 96-well plates, 100 µl of the medium containing triton-X were added per well. For experiments conducted in other plate formats, volumes were adjusted based on the total area of the wells. Data showed in Fig. S8 corresponding to previous normalisation was done using cell survival via CCK-8 assay following the manufacturers' procedure. In short, cells were washed once with PBS and incubated for 2 h at 37°C, 5% carbon dioxide with 100 µl fresh medium containing 10 µl of CCK-8. After incubation, 100 µl were collected and absorbance was read at 460 nm wavelength using a SpectraMax M5 plate reader. Due to the CCK-8 assay dependence on mitochondrial activity, the displayed data in the main text of this manuscript correspond to normalisations generated via LDH assay.

### RNA extraction and cDNA conversion

Cells were detached with accutase and pellets were frozen dry at −80°C. Cell pellets were thawed on ice and RNA extraction was done using the RNeasy Mini Kit. Samples were DNAse treated RNAse-Free DNase Set. RNA concentration was quantified using a Nanodrop 2000c. RNA was reversed transcribed into complementary DNA (cDNA) using the High-Capacity RNA-to-cDNA™ Kit. For cDNA conversion, samples were diluted, and equal concentration of RNA was added for the reaction.

### Quantitative polymerase chain reaction

Gene expression was determined using the double stranded DNA binding dye SYBR™ Green. Primers efficiency of designed non-published primers was determined by SYBR green qPCR across 5×3-fold dilutions of cDNA and calculated in Microsoft Excel as *Efficiency* = 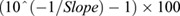 where slope is calculated for the plot of average Ct against Log(sample quantity) – see Fig. S7. qPCR reactions comprised of 1 volume of sample cDNA to 3 volumes mastermix (Power SYBR™Green PCR Master Mix, forward and reverse primer mix, and nuclease free water at a ratio of 5 µl:1 µl:1.5 µl, respectively). Reactions were run in five replicates in either 96-well format, 20 µl/sample, or 384-well format, 10 µl/sample. The threshold cycle (2^−ΔΔCt^) method of comparative PCR was used to analyse the results. 2^−ΔCt^ was calculated by the normalization of the sample Ct value to the average Ct value of two housekeeper genes, UBC and TBP. 2^−ΔΔCt^ analysis was done by the further normalisation of the samples against a 0 h control. For the quantification of ZIKV transcripts, Ct values were also normalised against UBC and TBP and further normalised against uninfected controls, displaying results as 2^−ΔCt^ and 2^−ΔΔCt^.

### Statistical analysis and software

Datasets from single experiments with large sample size, to compute for the time-course comparisons, were analysed by non-parametric paired Wilcoxon tests. Datasets containing missing values and/or values of zero after normalization from independent experiments of three donors' lines were analysed using mixed-effects model with Holm-Šidák correction. Datasets comprising two independent repeats of a donor patient line were analysed by two-tailed Mann–Whitney *U*-test. Datasets containing analysis of two independent donors or independent triplicate experiments of one patient line were analysed using mixed-effects model with Šidák correction. Datasets comprising one repeat of two patients' lines were analysed by mixed-effects model with Tukey's multiple comparisons test. Datasets containing analysis of independent experiments of three patients' lines comprising two groups were processed using two-way ANOVA with Šidák's multiple comparisons post-hoc test whilst when comprising three or more groups post-hoc were computed using Tukey's multiple comparisons test. All the statistical tests were performed using GraphPad Prism 9.3.1. A calculated *P*-value less than 0.05 was reported as significantly different. Representation schemes were created with BioRender.com. Figures were done using GNU Image Manipulation Program (GIMP 2.8.22).

## Supplementary Material

10.1242/biolopen.059889_sup1 Supplementary information
