# Supplementary material for: Zika virus-induces metabolic alterations in fetal neuronal progenitors that could influence in neurodevelopment during early pregnancy
Source: Biol Open. 2023 Apr 24;12(4):bio059889. doi: 10.1242/bio.059889 (PMC10151830; doi:10.1242/bio.059889)
Supplement: Supplementary information [file biolopen-12-059889-s1.pdf]

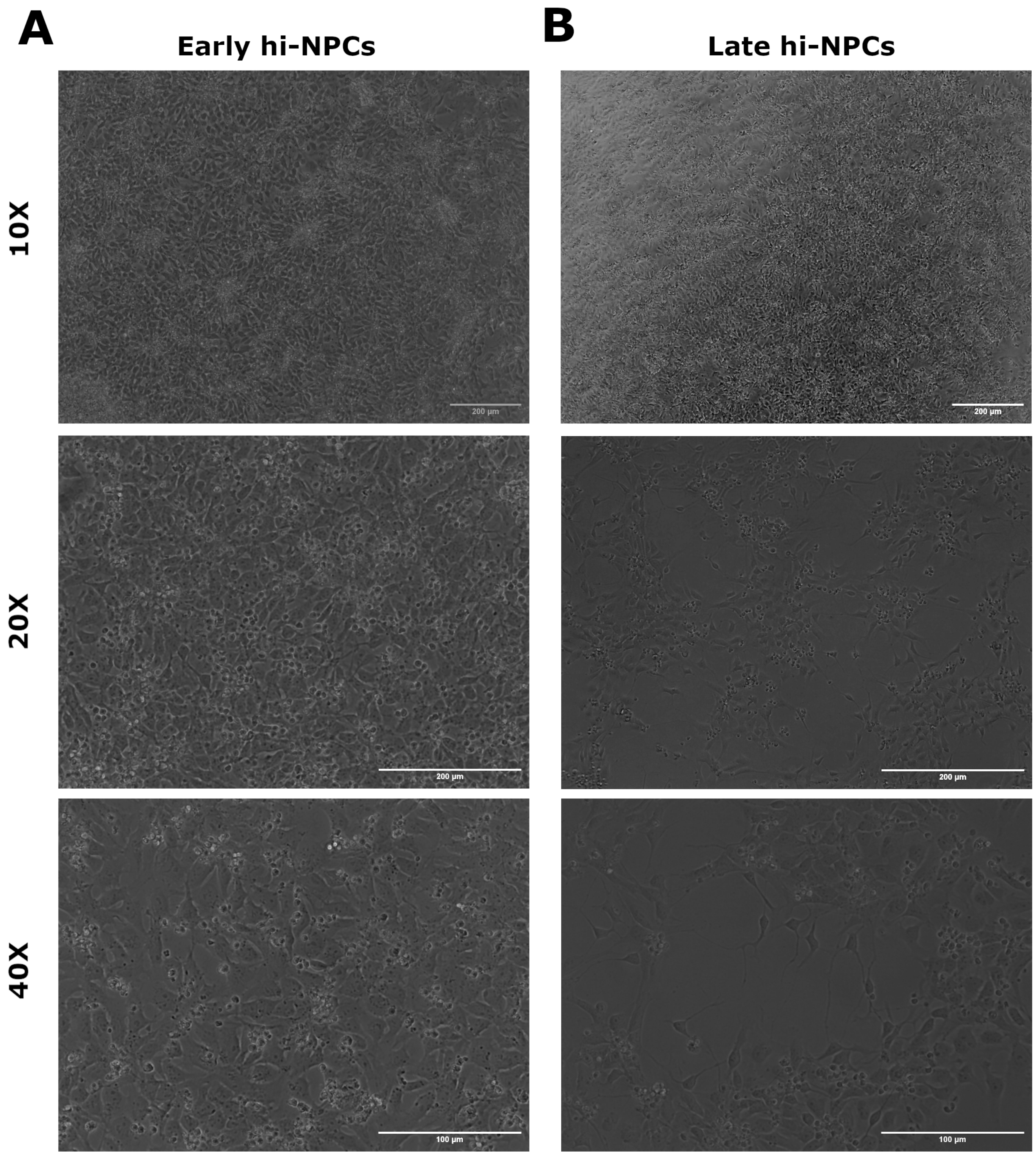

Fig. S1. Morphology of cells present within early and late hi-NPC cultures
Representative brightfield images of (A) early and (B) late hi-NPCs at different magnifications. Scale bar: 200 μm (10X and 20X) and 100 μm (40X).

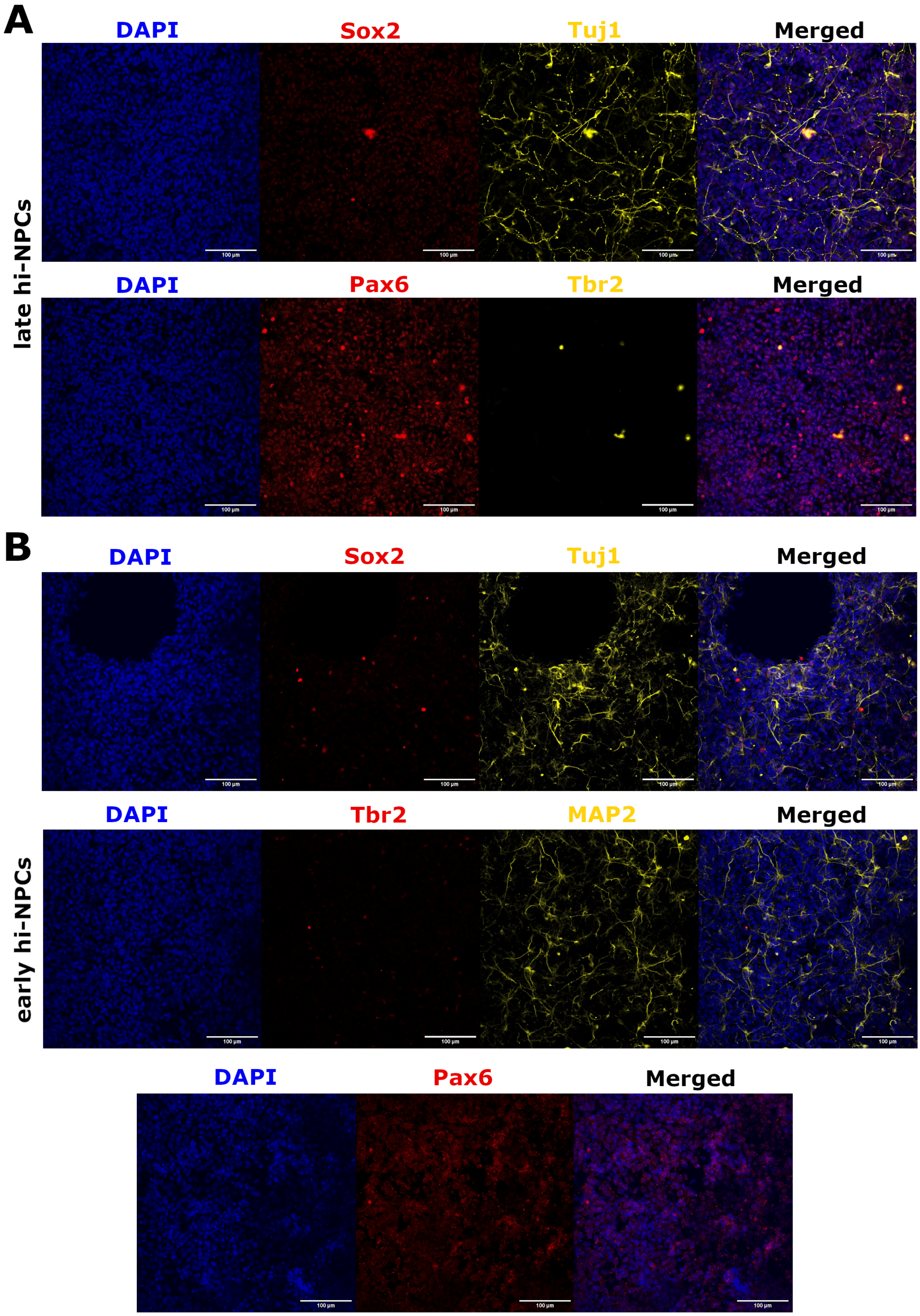

Fig. S2. Phenotypically distinct cortical neuronal progenitors differentiated from hiPSCs express markers of progenitors of the forebrain

Representative confocal images (10x) of the detection of markers of *in vitro* neuronal progenitors of the forebrain (Sox2, Tuj1, Tbr2, Pax6 and MAP2) in early and late hi-NPCs. Scale bar: 100 μm.

Biology Open • Supplementary information

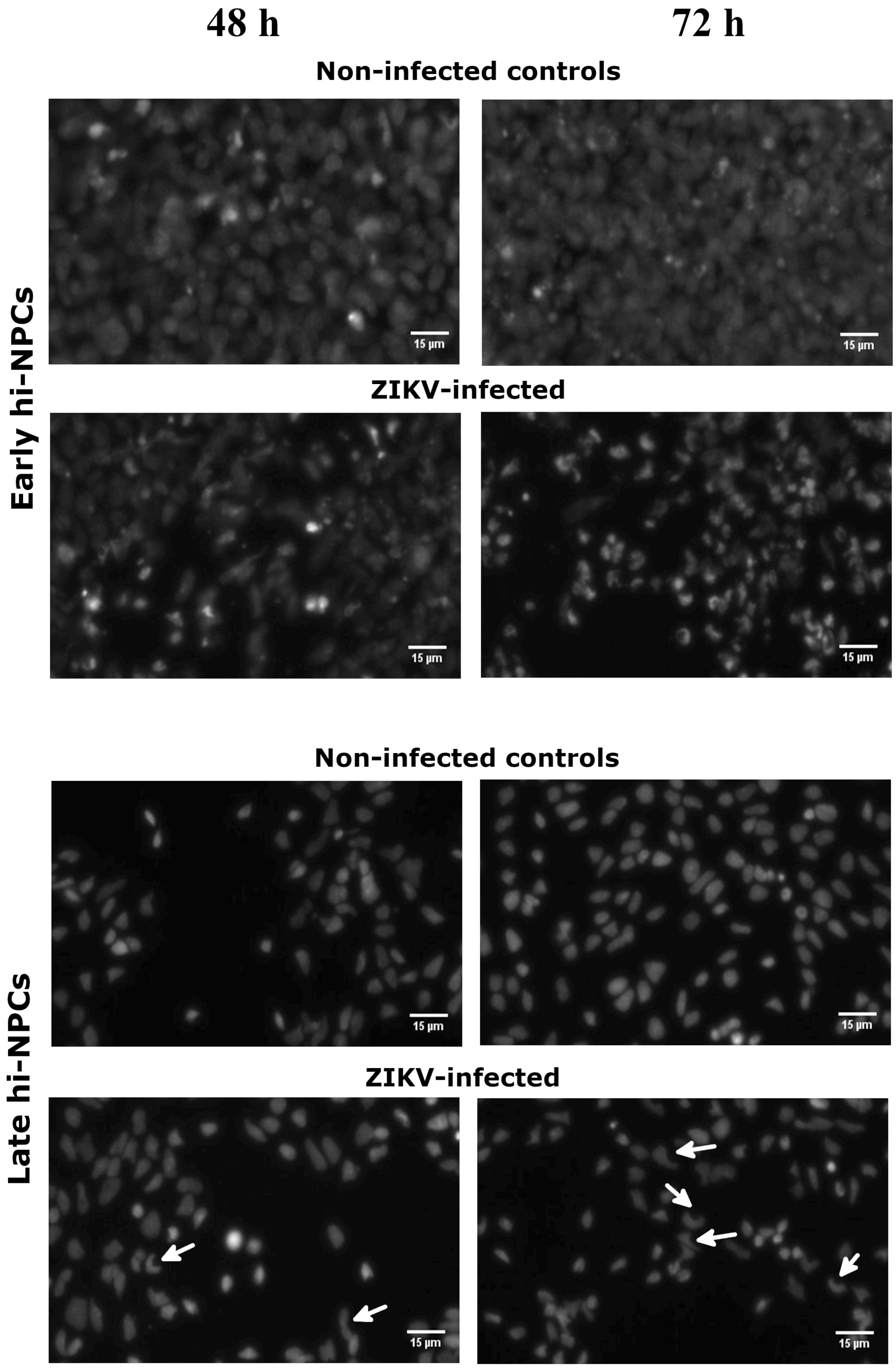

Fig. S3. ZIKV infection causes nuclear damage in early hi-NPCs

Imaging (40x) of the nuclei of early hi-NPCs infected to ZIKV over the course of 72 hours and the respective non-infected controls. Nuclei were stained with DAPI.

Biology Open • Supplementary information

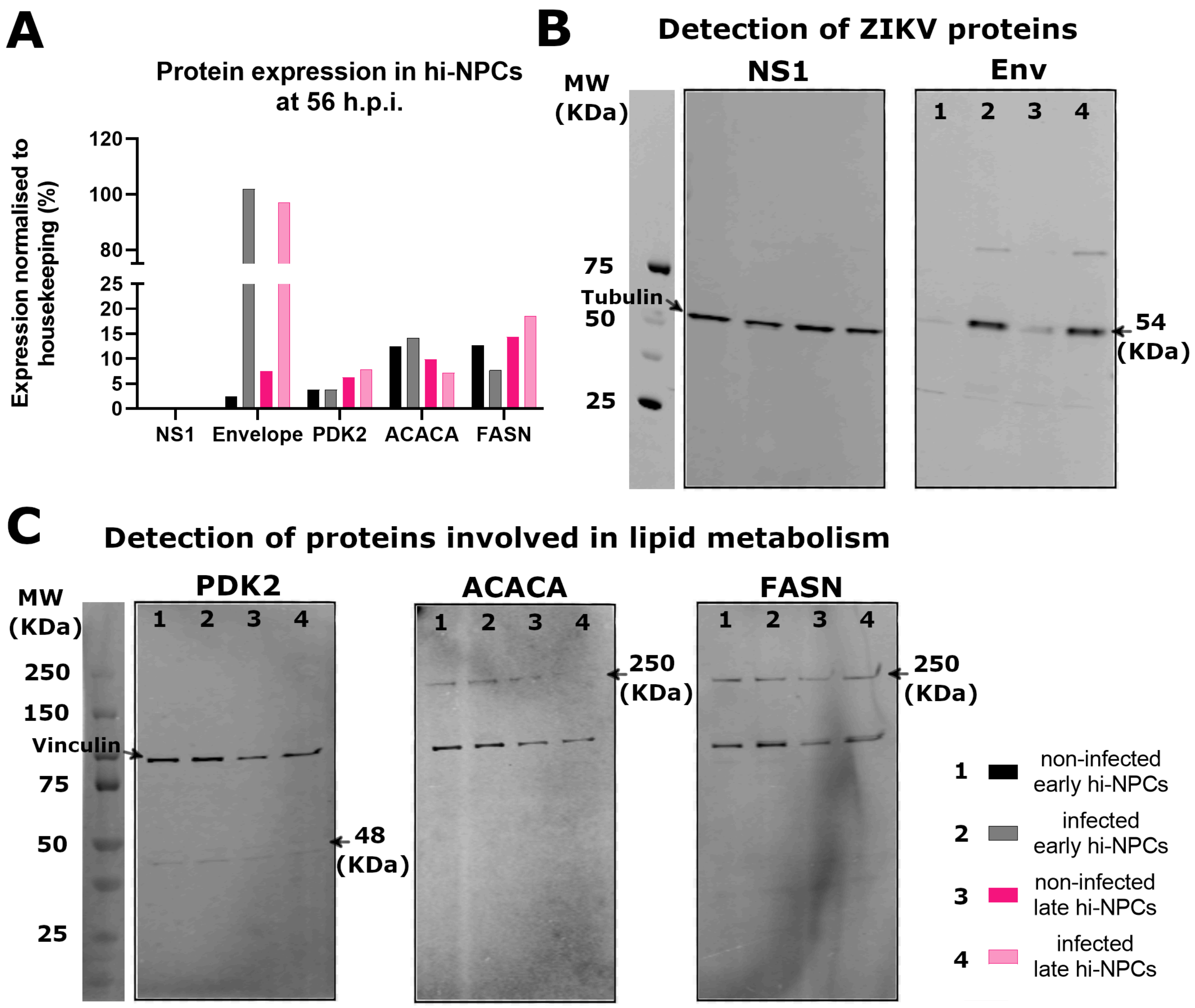

Fig. S4. Protein detection in non-infected and infected hi-NPCs

(A) Bar graph showing the expression levels of several proteins (western blot) in non-infected and infected hi-NPCs normalised to the housekeeping controls. Raw data used to generate the bar graph is displayed in B and C. (B) Images showing the revealed western blots for ZIKV proteins NS1 and Envelope (Env). (C) Images showing the revealed western blots for proteins involved in lipid metabolism (left to right, PDK2, ACACA and FASN).

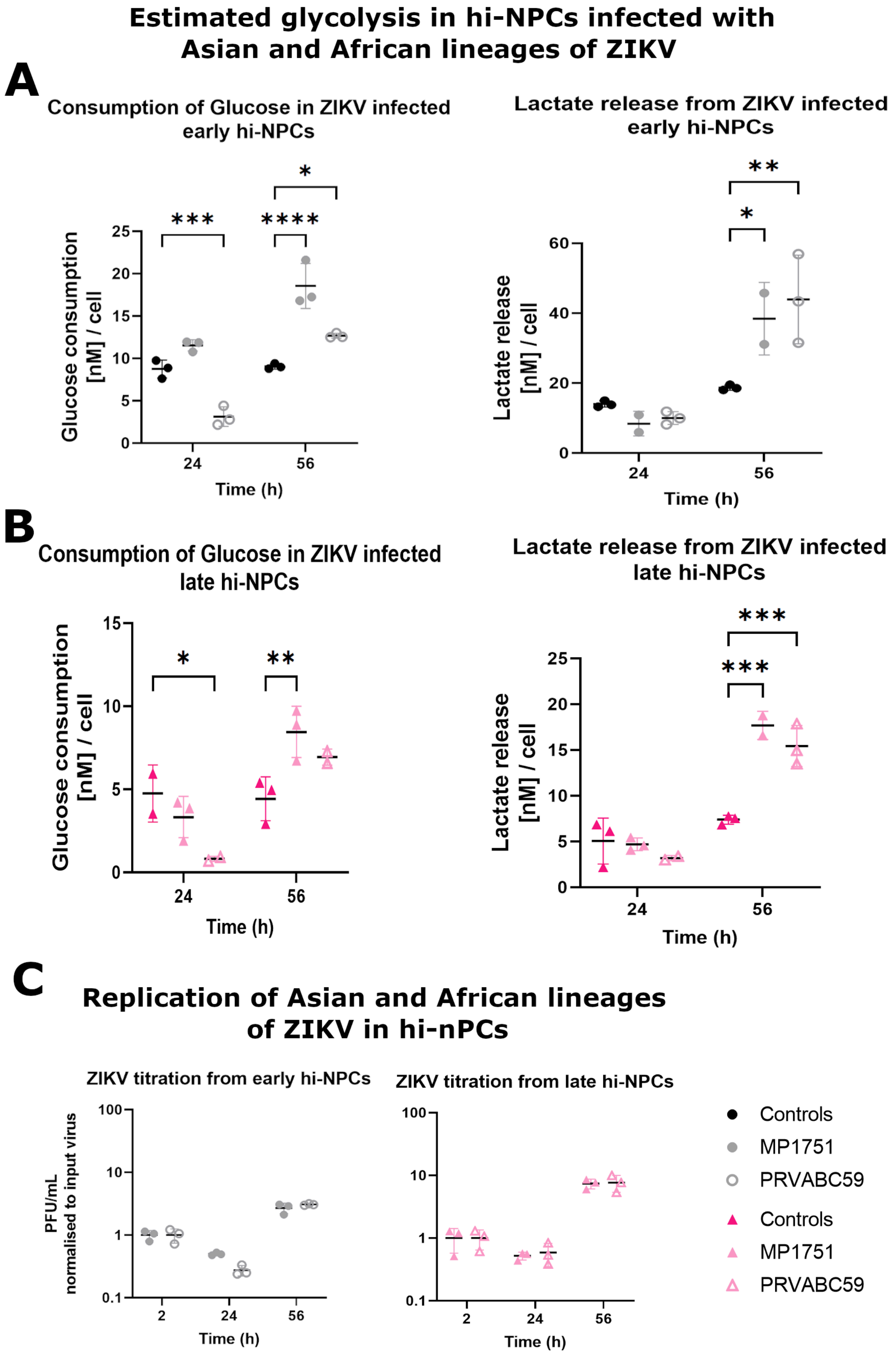

Fig. S5. Replication of African and Asian lineages of ZIKV in hi-NPCs induces similar alterations in their glycolytic capacity

The estimated glycolysis was assessed by the calculated glucose consumption and lactate release from ZIKV-infected and non-infected (A) early hi-NPCs and, (B) late hi-NPCs. (C) Titration of ZIKV strains PRVABC59 and MP1751 in early and late hi-NPCs normalised to input virus (MOI 1). Dots displaying the values of each patient line n = 1. Single viral infection was conducted in three independent patients' lines. ZIKV titration was done in triplicates from each sample whilst glucose and lactate measurements were conducted in duplicates. Significance was calculated by mixed-effects model with Holm-Šidák correction for the analysis of glucose and lactate. Significance was calculated by two-way ANOVA with Šidák's multiple comparisons test for ZIKV titration. Error bars display mean ± SD. Significance is shown when *$p < 0.05$, **$p < 0.01$, ***$p < 0.001$.

Biology Open • Supplementary information

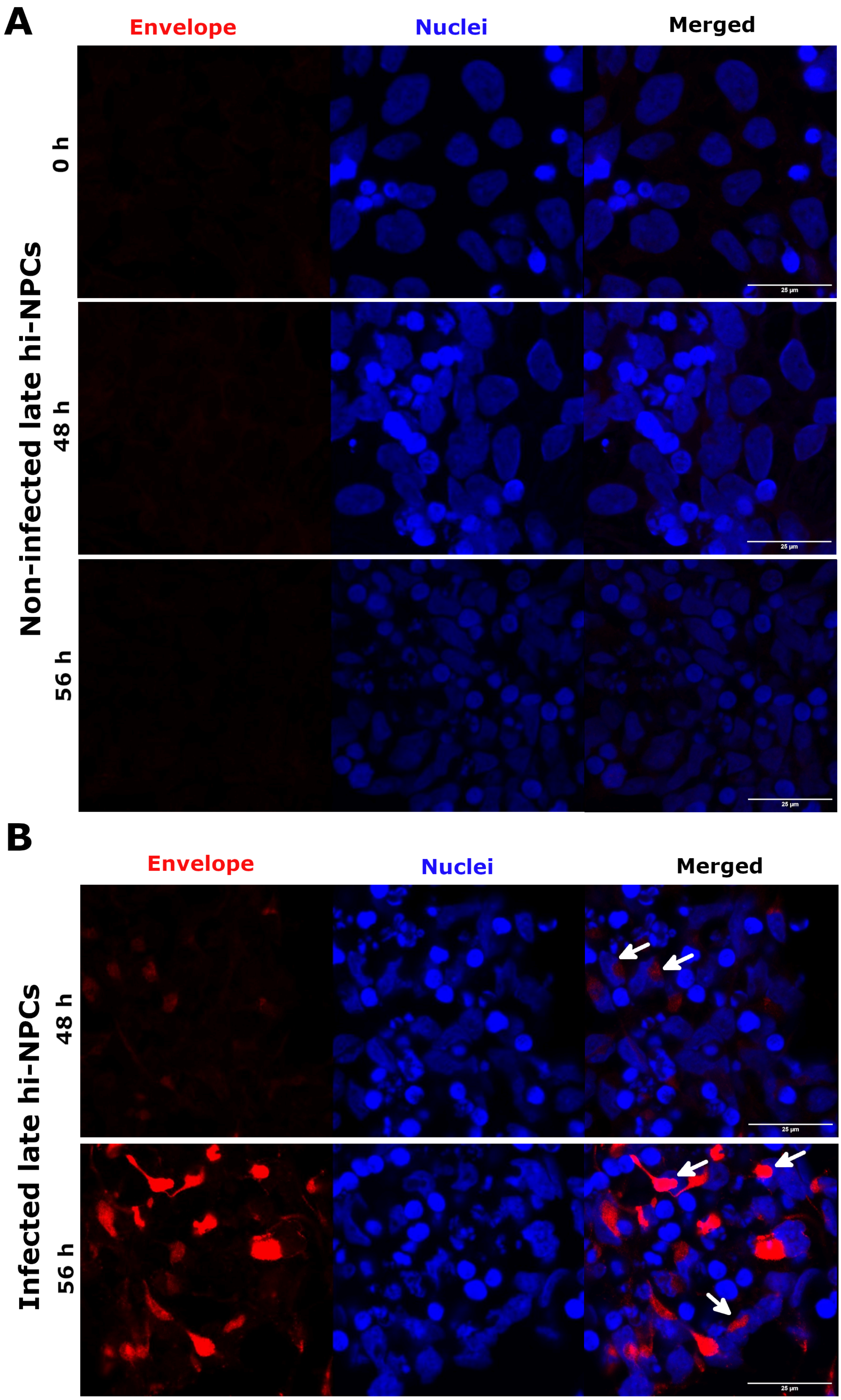

Fig. S6. ZIKV localises perinuclear and cytoplasmic in late hi-NPCs

Representative confocal images (63x) of the detection of ZIKV in late hi-NPCs. (A) Non-infected late hi-NPCs controls showing background detection of Envelope protein (red) and nuclear morphology (DAPI – blue). (B) Early stages during the replication kinetics of ZIKV show accumulation of Envelope protein (red) in perinuclear compartments (white arrows) with abundant cytoplasmic distribution at later stages. Scale bar: 25 µm.

Gene	Forward Primer	Reverse Primer	Efficiency (%)
ZIKV	AGGATCATAGGTGATGAAGAAAAGT	CCTGACAACACTAAGATTGGTGC	102.19
PDK-2	GCCGCTTGGATCTTTGTGGA	CCAGCTCCTTCTATCCTCCCTT	121.94
UCP2	GTCCGGTTACAGATCCAAGGAG	GGCAACCAGCCCATTGTAGA	96.57
HK-1	GCAGCTCCTGGCCTATTACT	TGGCATAGAGATACTTGTCAATCT	86.63
ACACA	ACCTGTAGTAGCTGCTGGTG	TCCAGGGCATACTTGGTAGC	102.02
HADHA	CCAGAAGATAGTGGACCGGC	AGGGCTGTTAGCATGGTCAG	101.12
FASN	GTCTCTGAAGGGCATCCTGG	GAGACAGTTCACCAAGCCCA	111.14
ACADM	GTGGAAGCAGATACCCCAGG	GCAGCTACTACAGGTCTGGT	102.10

Fig. S7. Efficiency and sequence of primers

Figure showing the sequence and calculated efficiency of the unpublished primers designed to assess the metabolic transcriptional profile of hi-NPCs during exposure to ZIKV. Primers were concentrated at 100 nM and the annealing temperature at 64 °C.

Biology Open • Supplementary information

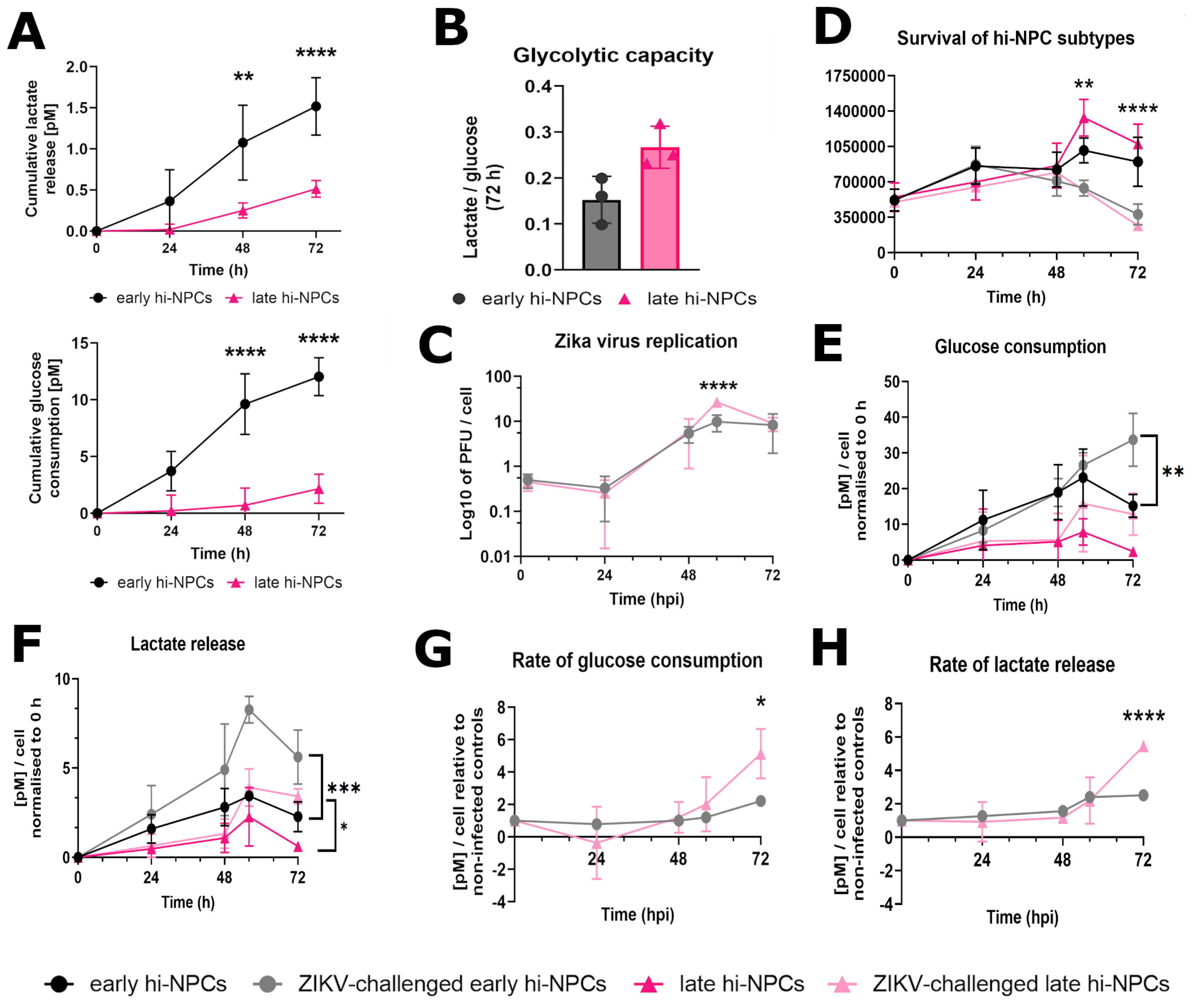

Fig. S8. Data normalisation based on tetrazolium salts may reflect greater errors in data analysis due to the dependence on mitochondrial health

(A) Dot plots showing the estimated cumulative lactate release and glucose consumption in hi-NPCs cultured over 72 h. (B) Bar graph displaying the glycolytic capacity of each hi-NPC at 72 h post-culture. Dot plots showing the (C) replication kinetics of ZIKV in early and late hi-NPCs and (D) the survival of each culture during ZIKV infection compared to non-infected controls. Dot plots showing (E) the consumption of glucose, (F) the release of lactate and, (G-H) the estimated rate of aerobic glycolysis in infected hi-NPCs compared to non-infected controls. Significance was calculated by Two-way ANOVA with Šidák's multiple comparisons post hoc test. Error bars display mean ± SD. Significance is shown when *p < 0.05, **p < 0.01, ***p < 0.001, ****p < 0.0001. Error bars display mean ± SD. Significance is shown when *p < 0.05, **p < 0.01, ***p < 0.001, ****p < 0.0001.
